# The Effects of Online Working Memory Training on Enhancing Hedonic Processing in People With Social Anhedonia and Subsyndromal Depression: An Exploratory Study

**DOI:** 10.1002/pchj.70084

**Published:** 2026-02-09

**Authors:** Jie Pu, Qian Ren, Yi‐hang Huang, Xuan Wang, Ling‐ling Wang, Hui‐xin Hu, Yi‐jing Zhang, Yun‐ru Wang, Yi Wang, Jia Huang, Ya Wang, Simon S. Y. Lui, Raymond C. K. Chan

**Affiliations:** ^1^ Neuropsychology and Applied Cognitive Neuroscience Laboratory, State Key Laboratory of Cognitive Science and Mental Health Institute of Psychology, Chinese Academy of Sciences Beijing China; ^2^ Department of Psychology University of Chinese Academy of Sciences Beijing China; ^3^ School of Psychology Shanghai Normal University Shanghai China; ^4^ Department of Psychology, School of Humanities and Social Sciences Beijing Forestry University Beijing China; ^5^ National Institute on Drug Dependence and Beijing Key Laboratory of Drug Dependence Peking University Beijing China; ^6^ Peking University Sixth Hospital, Peking University Institute of Mental Health, NHC Key Laboratory of Mental Health (Peking University), National Clinical Research Center for Mental Disorders (Peking University Sixth Hospital) Chinese Academy of Medical Sciences Research Unit (No. 2018RU006), Peking University Beijing China; ^7^ School of Psychology Capital Normal University Beijing China; ^8^ Department of Psychiatry, School of Clinical Medicine The University of Hong Kong Hong Kong Special Administrative Region China

**Keywords:** anhedonia, cognitive remediation, subclinical population, working memory training

## Abstract

Working memory (WM) training is considered a promising cognitive remediation for psychopathological disorders. Given the shared neural circuits in WM and hedonic processing, as well as the positive findings in schizophrenia patients with anhedonia, we hypothesized that WM training might improve hedonic processing in subclinical individuals. This study investigated the transfer effect of a 10‐session WM training on (1) people with social anhedonia and (2) people with subsyndromal depression, relative to the control groups. We evaluated the impact on different dimensions of anhedonia. A total of 152 Chinese university students were enrolled, and the study examined the general improvement of the hedonic training across trait and control groups. Findings showed that WM training improved the engagement of difficult tasks in participants with social anhedonia and the pleasure after paying effort in participants who had WM growth during the training sessions. The transfer effects on reward processing and cost–benefit computations indicated the benefits of WM training effects. Results were limited to subclinical samples within a short‐term intervention and might not generalize to clinical samples. In conclusion, our findings suggest that WM training could be a prospective cognitive remediation for alleviating anhedonia, warranting further exploration.

## 1 Introduction

Working memory (WM) is a crucial cognitive function that temporarily stores and manipulates information for complex tasks (Baddeley 1992, 2012). Impaired WM is observed in patients with schizophrenia (SCZ) and major depressive disorder (MDD; Forbes et al. 2009; Nikolin et al. 2021; Wang et al. 2021; Wu and Jiang 2020), and is associated with impaired hedonic processing (Gold et al. 2008). Empirical findings indicate that first‐episode SCZ patients with more severe negative symptoms exhibited more severe WM impairment (Chan et al. 2015). A five‐year longitudinal study suggested that WM was associated with negative symptoms and treatment outcome in first‐episode psychosis patients (González‐Ortega et al. 2013). Moreover, WM‐related functional connectivity of the brain predicted negative symptoms at an 79% accuracy (Nejad et al. 2013). WM influenced the emotional response to positive stimuli, perhaps related to its effects on anticipatory pleasure (Burbridge and Barch 2007; Gold et al. 2008) and the ability to integrate prior pleasure with current situations (Horan et al. 2006).

Several theoretical models have proposed how WM influences hedonic processing (Chan et al. 2022; Gooding and Pflum 2022; Kring and Barch 2014). Gold et al. (2008) suggested that WM is essential for retrieving prior memory with motivational salience, prospecting future events, and anticipating pleasure. Consequently, impaired WM could undermine one's motivation and goal‐directed behaviour. In Baddeley (2021)'s WM model, the episodic buffer integrates different information from various sources, for conscious information processing, including hedonic processing. WM may function to maintain the mental representation of reward over time to motivate subsequent behaviours. Neurobiologically, WM and hedonic processing both involve the frontal‐striatal network (Parr et al. 2020; Smith and Berridge 2007; Wang et al. 2021). It is plausible that cognitive impairments and negative symptoms share common neural substrates (Li et al. 2015).

Cognitive remediation is a useful intervention for SCZ‐associated cognitive impairments (Cella, Preti, et al. 2017; McGurk et al. 2007). Meta‐analysis supports the finding that cognitive remediation could yield small‐to‐moderate effects in reducing negative symptoms of SCZ (Cella, Stahl, et al. 2017). Moreover, WM training can promote neural activation, which in turn may reduce negative symptoms and enhance social motivation in SCZ patients (Li et al. 2019). In fact, activation likelihood estimation (ALE) meta‐analysis suggested that WM training may promote ‘neural plasticity’ in SCZ patients and healthy people (Li et al. 2015). For MDD, few studies have investigated the effects of WM training on anhedonia, yet previous studies found improvements in depressive symptoms and reduced ruminations after WM training (He et al. 2021; Ronold et al. 2022). In people with social anhedonia, who are at high risk of developing SCZ, WM training enhanced neural activations for better anticipation (Li, Li, et al. 2016). In people with subsyndromal depression, who are at risk of developing MDD, WM training enhanced self‐report pleasure and laboratory‐based motivation (Zhang et al. 2019). However, several studies reported mixed results (Cella, Preti, et al. 2017; Hou et al. 2023), hence further work is needed to clarify the effectiveness of WM training on anhedonia.

Anhedonia, a complex construct comprising multiple sub‐processes, includes prospection, reward processing, cost–benefit computation, and belief updating (Chan et al. 2022; Gold et al. 2008; Kring and Barch 2014). Previous studies on the transfer effects of WM training suggested that the approach sensitivity to affective rewards and prediction‐related neural activations in subclinical samples with high social anhedonia could be improved after training (Li, Li, et al. 2016; Li, Xiao, et al. 2016). Similar effects were reported in SCZ patients with high negative symptoms and people with subthreshold depressive symptoms (Li et al. 2019; Zhang et al. 2019). Given that anhedonia is multifaceted, a single behavioural task is insufficient to capture the transfer effects of WM training. A diminished WM capacity may lead to rapid decay of pleasure, and disabling individuals to effectively evaluate the cost of effortful actions. However, to date, very few studies on effects of WM training have adopted a multi‐dimensional approach to clarify the changes of multiple hedonic processes.

This study aimed to explore the potential transfer effects of WM training on the different hedonic processes, including (1) reward processing, (2) prospection, and (3) cost–benefit computation, in people with social anhedonia and people with subsyndromal depression. We administered a standardised, 10‐session online WM training program conducted within 2 weeks to all participants and measured WM at baseline and after the training using sophisticated behavioural tasks which are designed to capture the hedonic processes. Drawing on the theoretical framework that WM provides the cognitive scaffolding for reward‐related information, we propose that expanding WM capacity may facilitate more robust mental representations of rewards and enhance the integration of reward‐cost information. This, in turn, should alleviate the motivational deficits of subclinical social anhedonia and subsyndromal depression. Consequently, we hypothesized that (1) people with social anhedonia, people with subsyndromal depression, and healthy people would all benefit from WM training in terms of enhanced reward processing, prospection, cost–benefit computation. Furthermore, considering that healthy controls may exhibit a ceiling effect, whereas subclinical groups possess greater ‘room for improvement’ in their neural efficiency of reward representation, we also anticipated that (2) the subclinical groups would benefit more from WM training than the healthy control group.

## 2 Methods

### 2.1 Participants

Our sample originated from a large sample of another study which investigated the mental health of Chinese university students using questionnaires tapping into social anhedonia and subsyndromal depressive symptoms. Eligibility criteria included (1) an estimated IQ > 80; and (2) no personal or family history of neuropsychiatric disorders, and (3) no personal history of neurological disorders or substance abuse. In this study, participants completed the Chinese version of the Chapman Social Anhedonia Scale (CSAS; Chan et al. 2012) for assessing social anhedonia traits, the Beck Depression Inventory (BDI; Zheng and Lin 1991) and the Patient Health Questionnaire (PHQ; Wang et al. 2014) for subsyndromal depressive symptoms. Participants were categorized into four subgroups (Beck et al. 1988; Kroenke et al. 2001; Wang et al. 2014), namely the subgroup with high social anhedonia (SA; CSAS score ≥ 20, PHQ score < 10, and BDI score < 10), the subgroup with subsyndromal depression (SD; CSAS score < 20, PHQ score ≥ 10, or BDI score ≥ 10), the subgroup with co‐occurrence of high social anhedonia and subsyndromal depression (CO; CSAS score ≥ 20, PHQ score ≥ 10, or BDI score ≥ 10), and the subgroup with neither social anhedonia nor subsyndromal depression (CN; CSAS score < 20, PHQ score < 10, and BDI score < 10). Based on the cut‐off scores of the CSAS and PHQ, we recruited 47 participants with SA, 75 participants with SD, 67 participants with CO, and 70 participants with CN, see Table 1 for demographics of the final analytical sample. The training and measurements were conducted online using the *Pavlovia* platform (https://pavlovia.org/) (Peirce et al. 2019; Sauter et al. 2020).

**TABLE 1:** Demographic information and baseline performances of all participants.

	Social Anhedonia (*n* = 31)	Subsyndromal Depression (*n* = 42)	Co‐occurrence (*n* = 37)	Controls (*n* = 42)	*F*/*χ* ^2^	*p*	*η_p_* ^2^/*φ*
Age (year)	20.39 (1.48)	20.62 (1.71)	20.86 (2.88)	20.45 (2.01)	0.372 (3, 148)	0.773	0.007
Education level (year)	14.19 (1.87)	13.98 (1.56)	14.41 (1.54)	13.69 (1.33)	1.496 (3, 148)	0.218	0.029
Gender (male%)	10.00%	29.00%	27.00%	19.00%	0.204 (3)	0.204	0.174
Estimated IQ	128.42 (10.71)	129.1 (7.61)	125.27 (12.43)	130.45 (8.16)	1.95 (3, 147)	0.124	0.038
Logical memory (immediate)	15.00 (5.00)	14.05 (3.7)	13.54 (3.74)	15.57 (3.88)	1.996 (3, 148)	0.117	0.039
Logical memory (Delay)	13.81 (4.66)	11.26 (3.88)	12.08 (4.64)	13.86 (4.03)	3.555 (3, 148)	**0.016**	0.067
Visual reproduction (immediate)	23.16 (1.68)	22.14 (1.96)	22.41 (2.42)	23.33 (1.48)	3.589 (3, 148)	**0.015**	0.068
Visual reproduction (delay)	22.77 (2.12)	21.05 (3.18)	22.35 (2.29)	23 (1.23)	5.737 (3, 148)	**< 0.001**	0.104
Verbal fluency	26.45 (7.61)	24.31 (5.53)	24.89 (6.39)	26.57 (6.84)	1.156 (3, 148)	0.329	0.023
BDI	4.90 (3.10)	16.98 (7.1)	20.35 (8.31)	3.31 (3.22)	77.939 (3, 148)	**< 0.001**	0.612
PHQ‐9	4.90 (2.60)	10.86 (4.36)	13.22 (5.49)	4.19 (2.7)	46.63 (3, 148)	**< 0.001**	0.486
CSAS	23.61 (3.36)	12.9 (4.53)	25.62 (4.5)	5.88 (2.46)	226.886 (3, 148)	**< 0.001**	0.821

*Note:* Bold values indicates statisitcal significance at the *p* < 0.05 level.

Abbreviations: BDI: Beck Depression Inventory; CSAS: Chapman Social Anhedonia Scale; PHQ‐9: Patient Health Questionnaire‐9.

**FIGURE 1:**
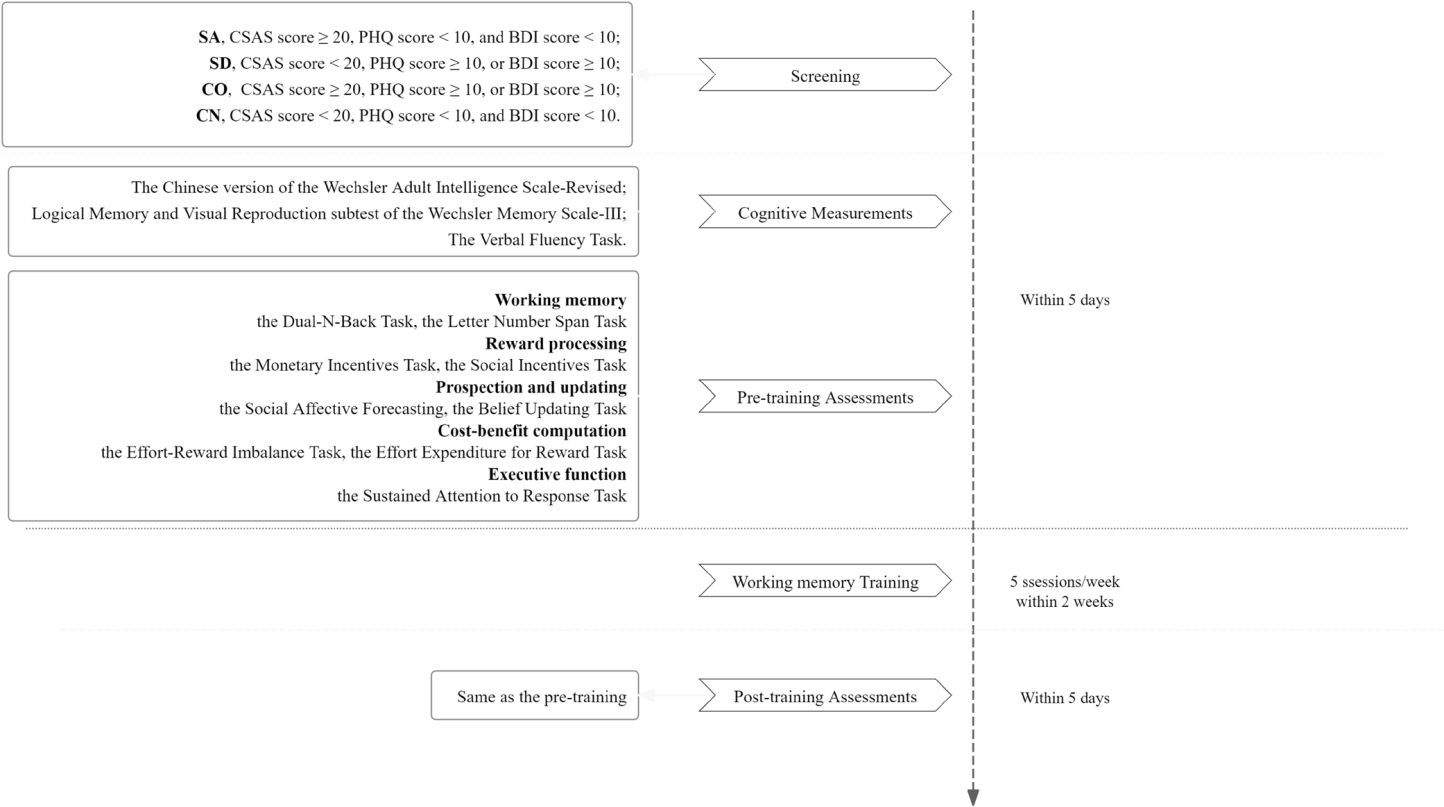
Procedures flowcharts.

This study was approved by the Ethics Committee of the Institute of Psychology, Chinese Academy of Sciences (H22106), and pre‐registered on the Chinese Clinical Trial Registry (ChiCTR2200065126). All participants provided written informed consent. Monetary incentive was provided upon completion of the WM training, and baseline and end‐point WM assessments (Figure 1).

### 2.2 WM Training

The adapted dual‐n‐back task was administered online, adapted from Jaeggi et al. (2008)'s original task, and has been applied to SCZ patients (Li et al. 2019), people with social anhedonia (Li, Li, et al. 2016), and people with subsyndromal depression (Zhang et al. 2019). Details of this task have been described elsewhere (Li, Li, et al. 2016). Participants were asked to determine whether the current visual and auditory stimulus was consistent with that of n trials back, and the presentations of both modalities are independent from each other. The visual target was presented in one of the 3 × 3 grids, while the auditory target was the sound of Chinese characters from one of the family names introduced before the run. Each block consisted of 20 + *n* trials, comprising 6 trials of visual target and 6 trials of auditory target. Each session consisted of 20 blocks, lasting for 20–30 min. The value of n in each block would increase by one when the correctly detected trials are > 83%, or decrease by one when the correctly detected trials are < 34%. Otherwise, it would remain the same. All sessions started with 1‐back. Auditory feedback was presented for correct identification of the target. The average of the n in each session was recorded as N‐average, and the maximum of the n in each session was recorded as N‐max, tracing the training progress along the course. Participants were asked to take one session of WM training each day as far as possible, and to avoid taking late‐night training. After five training sessions, participants were allowed to have a two‐day break before starting another 1‐week cycle of training. All training sessions must be completed within 2 weeks. The recruitment procedure is detailed in Supporting Information: Appendix A.

### 2.3 Cognitive Measures

Prior to WM training, we estimated participants' IQ, based on the four‐subtest of the Chinese version of the Wechsler Adult Intelligence Scale‐Revised (Gong 1992, 2). They also completed the logical memory and visual reproduction subtests of the Wechsler Memory Scale‐III (Tulsky et al. 2003), and the Verbal Fluency Task.

We administered the letter number span (LNS) task (Chan et al. 2008) and the dual‐n‐back task (Li et al. 2019) for WM capacity measure. The latter task contained a set of auditory materials (audio recordings of single‐character color names) which differed from that used in WM training. During the LNS task, participants were asked to re‐sequence the numbers and characters. We calculated the longest category passed (LNS‐longest) and the total number of accurate responses (LNS‐total) of LNS.

During the dual‐n‐back task, the session ends when the correction rate falls below 80% in either the auditory or visual target trial within block. We documented the average and maximum number of N in a session. Moreover, we administered the Sustained Attention to Response Task (SART; Chan et al. 2009). Participants were instructed to respond to non‐target stimuli and to withhold their responses to target stimuli.

### 2.4 Hedonic Processing Measures

Following the multiple‐component model of anhedonia (Chan et al. 2022), we administered a set of behavioural tasks to comprehensively measure the different facets of hedonic processing, that is, reward learning and motivation, prospection, belief updating, cost–benefit computation.

For reward learning and motivation, the computerized Monetary Incentives Delay (MID) and Social Incentives Delay (SID) tasks (Chan et al. 2016; Pu et al. 2024, 2; Xie et al. 2014; Zhang et al. 2022) were used. Participants were required to respond when a target was presented and would receive different feedback based on the reward, punishment, or neutral cue. Participants self‐rated their anticipatory pleasure before the targets and consummatory pleasure after receiving the feedback.

For prospection and belief updating, we administered the Social Affective Forecasting (SAF) task (Zhang et al. 2020) and Belief Updating (BU) task (Hu et al. 2022). In the SAF task, participants imagined positive/negative social/non‐social events, 2 specific scenarios for each type to simulate. They then self‐rated the valence and arousal of anticipatory pleasure (i.e., emotion forecasted in the future events) and anticipated pleasure (i.e., emotion experienced when forecasting the future events), as well as the effort they would devote to fulfill the positive events and phenomenological characteristics of their prospection (sensory details; self‐referential thoughts; other‐referential thoughts; and communications). Details of the BU task have been described in our previous work (Hu et al. 2022). In short, participants were asked to report their predictions on the probability of certain events occurring to them before and after being informed of the ‘average’ probability of a large sample survey. They could update their beliefs on the occurrence of the events or maintain the original prediction depending on the informed probability, defined as good/bad news.

For cost–benefit computation, the Effort Expenditure for Reward Task (EEfRT; Huang et al. 2016; Pu et al. 2024; Treadway et al. 2009; Wang, Gong, et al. 2024) and the Effort Reward Imbalance task (ERI; Yan et al. 2023) were used. In the EEfRT task, participants were offered a choice between low‐effort and high‐effort tasks. The reward for low‐effort tasks was fixed at ¥5, while for high‐effort tasks, the reward varied randomly from ¥5.4 to ¥6.4 or from ¥5.4 to ¥9.4. After choosing the difficulty level, participants pressed the keyboard to reach the target line in a bar corresponding to their task choice. Upon receiving the results of whether they had achieved their choice, participants rated their consummatory pleasure. In the ERI task, participants were asked to report as to how much they wanted to do the task. They then performed six mental arithmetic operations, by adding eight when a red figure was displayed, or by subtracting eight when a blue figure was displayed. They then compared the results with the on‐screen figure. A correct answer would be rewarded with 5–8 tokens. Finally, participants were asked to provide an expected reward based on their performance and to rate their anticipatory pleasure. Upon receiving the outcome, participants were asked to report their consummatory pleasure. Detailed descriptions of these paradigms can be found in Supporting Information: Appendix B.

### 2.5 Data Analysis

A linear model was fitted to characterize the training process, with the session maximum modeled as a function of the cumulative number of completed training sessions. The development achieved over the course of WM training would be reflected in the slope parameter. We excluded non‐responders in the primary analysis to examine the benefits of WM training, that is, participants who exhibited negative training performance slopes, in order to investigate the maximum potential transfer effect from WM training. To further explore variability in training response, we performed clustering analysis on the slope coefficients of all participants using the Ward's hierarchical clustering, based on the Euclidean distances. The analysis was conducted using the cluster and factoextra packages. The optimal number of clusters was determined by visually inspecting the dendrogram, supplemented by the average silhouette coefficient to assess the quality and stability of the resulting clusters.

ANOVAs and chi‐squared test were conducted to examine group differences in demographic characteristics. To assess training effects, we conducted repeated measures ANOVAs, with Group (SA/SD/CO/CN) as the between‐subjects variable, and Time‐Point (pre‐/post‐training) and Condition as the within‐subjects variables. Assumptions underlying repeated‐measures ANOVA were evaluated prior to statistical inference. Normality of residuals was examined through Q–Q plots, and no substantial violations were detected. Sphericity was assessed using Mauchly's test, and Greenhouse–Geisser corrections were applied where appropriate. Furthermore, to account for individual variability and to evaluate the robustness of our findings, we performed supplementary Linear Mixed Models (LMM) for all primary outcomes. In these models, Group, Conditions and Time‐Point were treated as fixed effects, with random intercepts included for each participant. Additionally, to address the potential overlap between subclinical trait constructs, we conducted a separate set of LMMs treating social anhedonia (CSAS) and depressive symptoms (BDI/PHQ) as continuous predictors. These supplementary results, which generally align with the ANOVA findings, are detailed in Supporting Information: Appendix C (Tables S4–S11). Table 2 shows the variables of experimental tasks. Specifically, in the MID and SID tasks, the Condition variable concerned the different feedback rules (reward/punishment/neutral). We recorded the reaction time, anticipatory pleasure, and consummatory pleasure in each trial. Any trials with a reaction time < 100 ms and hit trials < 6 in any of the three conditions would be deemed invalid and excluded.

**TABLE 2:** Summary of important variables in each task except for group and time point.

	‘Condition’ variables	Dependent variables
Hedonic processing		
Reward processing		
The Monetary Incentives Task	Reward: hit the target, get tokens as reward, miss the target, get nothing; Punishment: hit the target, get nothing, miss the target, lose tokens as punishment; Neutral: hit or miss, tokens remain unchanged.	Reaction time; Anticipatory pleasure; Consummatory pleasure when hit the target; Consummatory pleasure when miss the target
The Social Incentives Task	Reward: hit the target, get social approval expression as reward, miss the target, get calm expression; Punishment: hit the target, get calm expression, miss the target, get social disapproval expression as punishment; Neutral: hit or miss, get calm expression.	Reaction time; Anticipatory pleasure; Consummatory pleasure when hit the target; Consummatory pleasure when miss the target
Prospection		
The Social Affective Forecasting Task	Social Context/Nonsocial Context	Self‐reported anticipated emotion valence and arousal in positive/negative events; Self‐reported anticipatory emotion valence and arousal in positive/negative events; Self‐reported motivation to engage in positive events; Self‐rated details of the imaginings: (1) sensory details; (2) self‐referential thoughts; (3) other‐referential thoughts; and (4) communications.
The Belief Updating Task	Good News/Bad News: Estimation error (EE) was calculated by subtracting the base rate from the first estimation. In positive event trials, participants received ‘good news’ if the EE was positive and in negative event trials if the EE was negative, and vice versa for ‘bad news’.	BU score: the variance between the first estimation and the second estimation.
Cost–benefit computation		
The Effort Expenditure for Reward Task	Small reward range: 5.4–6.4 tokens Large reward range: 5.4–9.4 tokens	Decision proportion on high effort tasks; Consummatory pleasure rating after complete the chosen task; Range adaptability for reward on decision proportion; Range adaptability for reward on consummatory pleasure rating.
The Effort Reward Imbalance Task	Average: effort reward balance, actual reward equals to anticipated reward; Top: effort reward imbalance, actual reward is more than anticipated reward; Bottom: effort reward imbalance, actual reward is less than anticipated reward.	Wanting ratings of reward; Liking ratings of reward; Adaptability for imbalance on wanting ratings; Adaptability for imbalance on liking ratings.
Cognitive functions		
Working memory capacity		
Dual N‐Back Task		N‐max: the highest n‐back level in all blocks; N‐average: the average n‐back level of all blocks.
Letter Number Span Task		LNS‐max: the longest letter number span level passed in all the items; LNS‐sum: the total number of items passed.
Executive function		
The Sustained Attention to Response Task		Hit rate; Commission error

In the SAF task, analysis was conducted in both positive and negative contexts with social or non‐social features. We excluded participants who failed to complete all simulations. In the BU task, the Condition variable concerned the type of news (good/bad) given, with estimation error (EE) by subtracting the base rate from the first estimation. The update score was the variance between the first and the second estimation. Trials with zero estimation or incorrect entry (value beyond the range of 0%–100%) were excluded.

In the EEfRT task, linear regression modelling was applied to calculate the slope of the proportion of choosing high‐effort tasks and the slope of the consummatory pleasure ratings as functions of value. We also estimated the difference of slopes between the large and small reward amplitudes, respectively. In the ERI task, the effort‐reward condition (ratio = actual reward/anticipated reward) included effort‐reward balance (ERB, recorded as A, ratio = 1), effort>reward imbalance (E > *R*, recorded as B, ratio < 1), and effort<reward imbalance (E < *R*, recorded as T, ratio > 1), which was quantified by the difference between mental arithmetic accuracy and actual reward. The wanting or liking ratings and the beta of wanting or liking to assess the dynamic patterns of wanting and liking during changes in the effort/reward condition, which referred to adaptive changes in reward motivation, were examined. We only included participants who showed a mean accuracy of > 60% and included trials with a reaction time of > 200 ms. All interaction effects with respect to the time point were reported to better illustrate WM training effect. Detailed statistical analysis can be found in Supporting Information (see Appendix C: Tables S4–11). The significance level for the above analysis was set at *p* < 0.05.

## 3 Results

Thirty‐five SA participants, 45 SD participants, 39 CO participants, and 46 CN participants completed all of the training sessions required, corresponding to the completion rates of 74.47%, 60.00%, 58.21%, and 65.71% respectively. After exclusion of participants who did not exhibit positive training effects, the final data of 152 participants was entered for analysis (Mean_Age_ = 20.59 years, SD_Age_ = 2.08 years, 21.71% male). We acquired the training records from 31 SA participants, 42 SD participants, 37 CO participants, and 42 CN participants. The four groups were comparable in age (*F*(3,148) = 0.372, *p* = 0.773, *partial η* ^2^ = 0.007), estimated IQ (*F*(3,147) = 1.950, *p* = 0.124, *partial η* ^2^ = 0.038), education level (*F*(3,148) = 1.496, *p* = 0.218, *partial η* ^2^ = 0.029), and gender proportion (*χ* ^2^ = 0.204, df = 3, *p* = 0.204, *φ* = 0.174). All the results of training effects are summarized in Supporting Information (Table S12).

### 3.1 Linear Model Fitting of WM Training

**FIGURE 2:**
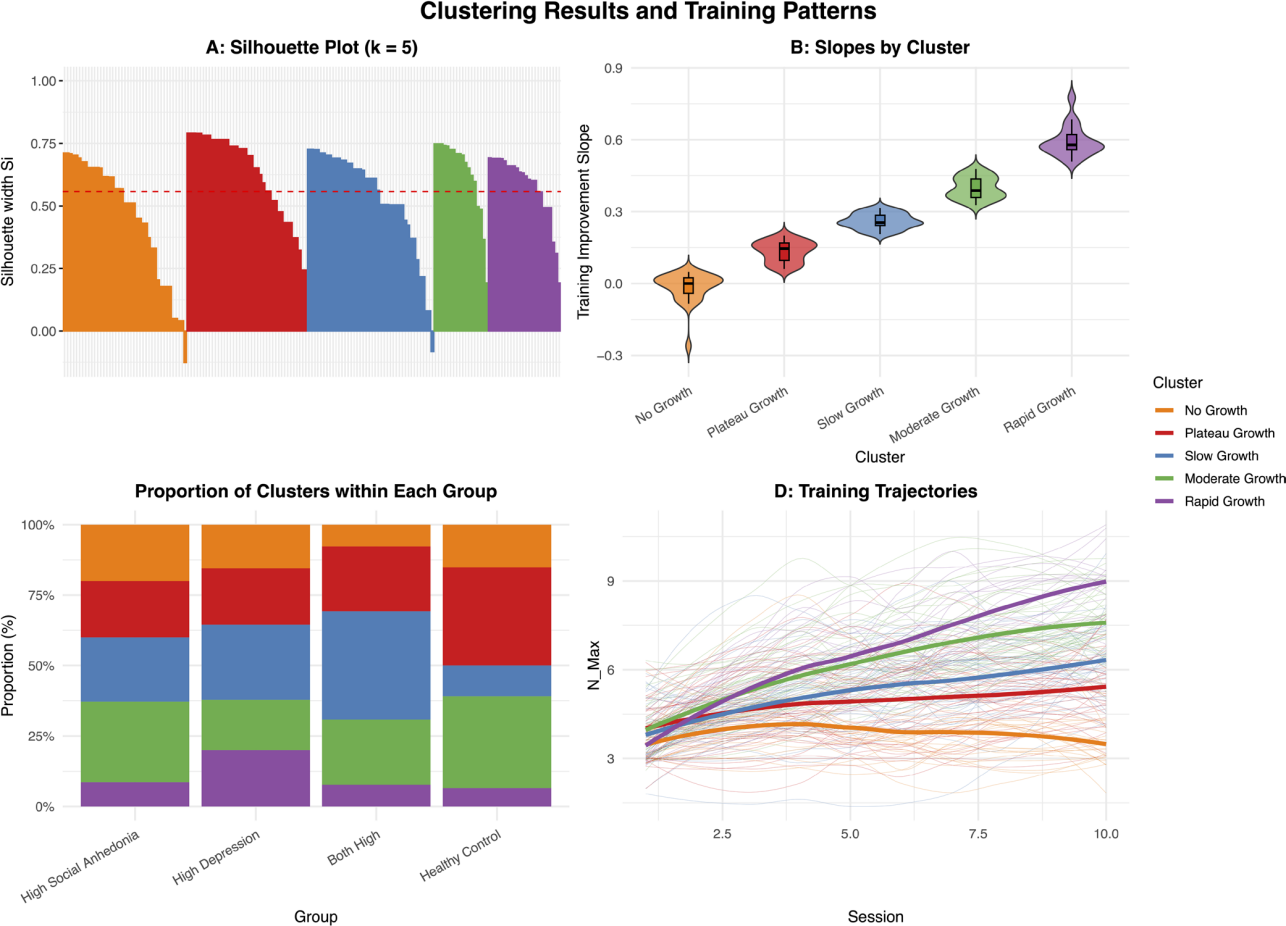
(A) Silhouette coefficient, with a mean value of 0.556. (B) Distribution of the training slopes within each cluster. (C) Proportion of clusters within each subclinical and control group. (D) Training trajectories for each cluster.

No group difference was found in the slopes of the fitted model for N‐max (*F*(3,148) = 0.587, *p* = 0.624, *partial η* ^2^ = 0.012) or N‐average (*F*(3,148) = 0.610, *p* = 0.610, *partial η* ^2^ = 0.012). As the training sessions increased, the amount of increase in WM capacity was equivalent (see Table S3). Notably, the number of participants who completed the pre‐ and post‐training measures for each of the paradigms (outcomes) varied, ranging from 22 to 41 in the subgroups (see Table S2). To complement the group‐based analysis and account for the full variability in training response, we performed a data‐driven clustering analysis on the individual training slopes, including all 162 participants. The hierarchical clustering (Ward's method) based on Euclidean distances of training slopes identified 5 distinct training patterns. The average silhouette coefficient was 0.556, indicating moderate cluster quality, ranging from ‘No Growth’ (negative or flat slopes) to ‘Rapid Growth’, and captured meaningful variation in training trajectories (see Figure 2).

### 3.2 Cognitive Gains

We observed a general improvement in WM capacity across all groups after WM training (*p* < 0.001). To control for the difference in baseline WM function, the standardized intercepts of the linear model for *N*‐max and *N*‐average fitted during WM training validation were included as the covariates. For the dual N‐back assessments, *N*‐max (*F*(1,146) = 132.086, *p* < 0.001, partial *η* ^*2*^ = 0.475) and N‐average (*F*(1,146) = 129.762, *p* < 0.001, partial *η* ^2^ = 0.471) showed a significant increase, but did not differ between groups (for the N‐max, *F*(3,146) = 1.443, *p* = 0.233, partial *η* ^2^ = 0.029; for the N‐average, *F*(3,146) = 1.470, *p* = 0.225, partial *η* ^2^ = 0.029). The Group‐by‐Time‐Point interaction effect (for the N‐max, *F*(3,146) = 1.430, *p* = 0.236, *partial η* ^2^ = 0.029; for the N‐average, *F*(3,146) = 1.425, *p* = 0.238, *partial η* ^2^ = 0.028) was not significant. We found significant improvement after WM training in both LNS‐longest (*F*(1,146) = 45.679, *p* < 0.001, *partial η* ^2^ = 0.238) and LNS‐total (*F*(1,146) = 77.157, *p* < 0.001, *partial η* ^2^ = 0.346). The Group main effect was significant for LNS‐longest (*F*(3,146) = 3.901, *p* = 0.010, *partial η* ^2^ = 0.074). Further simple effect analysis indicated a significantly lower score (*p* = 0.005) in the SD group than in the CN group after Bonferroni corrections. The Group main effect was not significant for LNS‐total (*F*(3,146) = 1.235, *p* = 0.299, *partial η* ^2^ = 0.025). The Group‐by‐Time‐Point interaction was not significant for either LNS‐longest (*F*(3,146) = 0.119, *p* = 0.949, *partial η* ^2^ = 0.002) or LNS‐total (*F*(3,146) = 1.842, *p* = 0.142, *partial η* ^2^ = 0.036) (see Table S4).

Regarding the SART, the reaction time (*F*(1,144) = 22.880, *p* < 0.001, *partial η* ^2^ = 0.137) was faster and the proportion of commission error (*F*(1,142) = 10.262, *p* = 0.002, *partial η* ^2^ = 0.067) reduced significantly after WM training. A ceiling effect was observed in the SART, thus the effect size for psychophysical approach was not efficient. Moreover, participants with no growth hit less correct (*p* = 0.001) after the sessions. Other results can be found in Table S11.

### 3.3 Hedonic Processing Improvement

**FIGURE 3:**
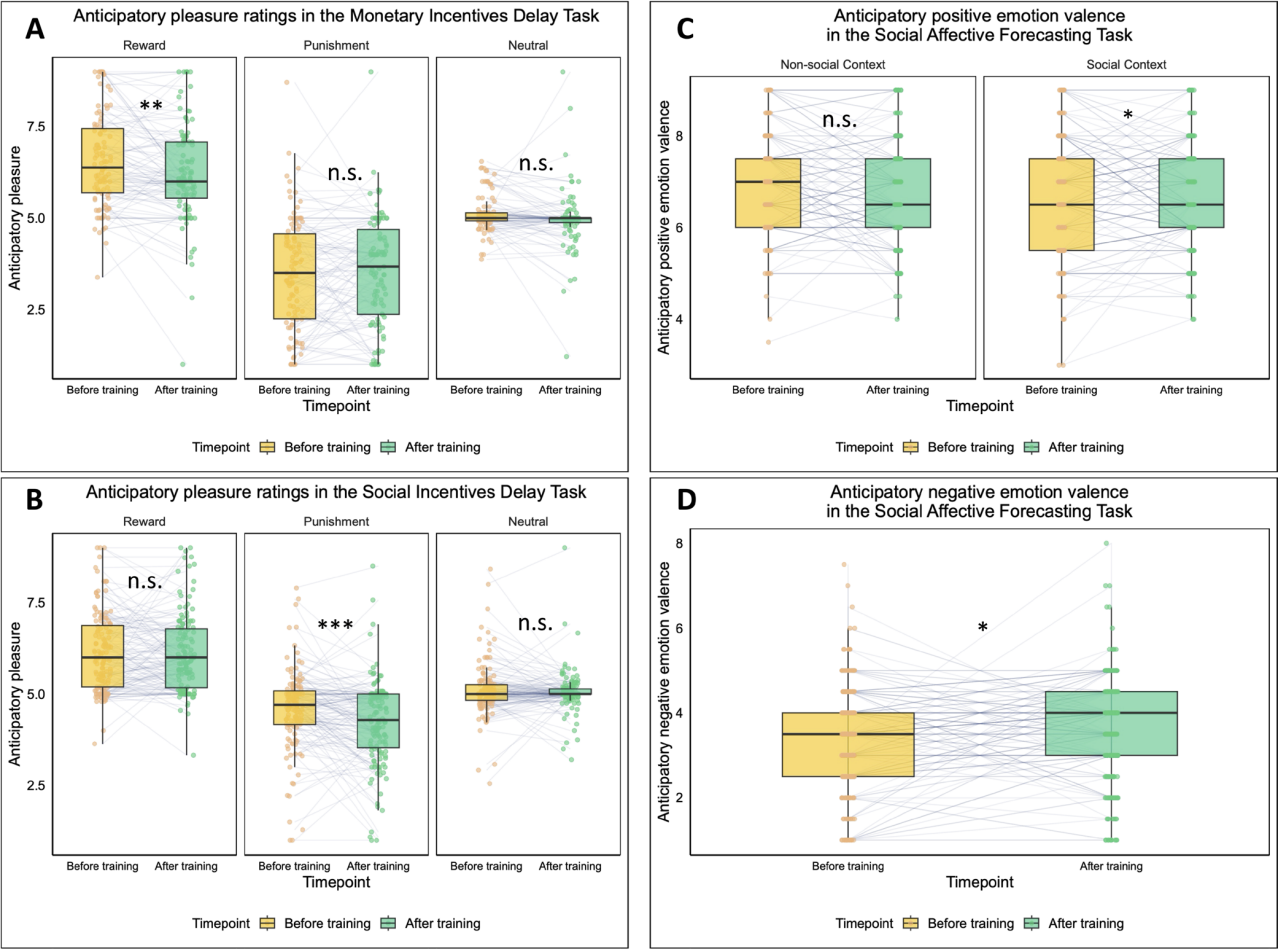
(A) Anticipatory pleasure with monetary feedback. In the reward condition, the anticipatory pleasure decreased significantly after the working memory training. (B) Anticipatory pleasure with social feedback. In the punishment condition, the anticipatory feeling decreased significantly after the working memory training. (C) Anticipatory emotion valence in positive events. A significant decrease in anticipatory positive emotion valence was investigated in non‐social contexts. (D) Anticipatory emotion valence in negative events. A significant increase in anticipatory negative emotion valence was investigated in non‐social contexts. *p < 0.05; **p < 0.01; ***p < 0.001; n.s., not significant.

Regarding reward processing and motivation, our results varied depending on the reward involved. The anticipatory pleasure decreased in the MID task (*F*(1,99) = 4.722, *p* = 0.032, *partial η* ^2^ = 0.046) and in the SID task (*F*(1,131) = 7.573, *p* = 0.007, *partial η* ^2^ = 0.055). The Condition‐by‐Time‐Point interaction was significant in the MID task (*F*(2,198) = 4.286, *p* = 0.026, *partial η* ^2^ = 0.041) as well as the SID task (*F*(2,262) = 4.948, *p* = 0.016, *partial η* ^2^ = 0.036). Further simple effects analysis showed that anticipatory pleasure decreased significantly only in the reward condition (*p* = 0.001), marginally significantly in the neutral condition (*p* = 0.065), and remained comparable in the punishment condition (*p* = 0.723) in the MID task (see Figure 3A and Table S5). However, in the SID task, anticipatory pleasure decreased significantly only in the punishment condition (*p* < 0.001), whereas it remained comparable in the reward (*p* = 0.689) and neutral (*p* = 0.396) conditions (see Figure 3B and Table S6). Notably, this alteration did not appear differently among participants with varied training growth (*p* = 0.933 for the MID, and *p* = 0.890 for the SID), indicating comparable changes across training trajectories.

Regarding prospection, as for the anticipatory emotion valence in positively forecasted events in the SAF Task, the Condition‐by‐Time‐Point interaction was marginally significant, *F*(1,140) = 3.089, *p* = 0.081, *partial η* ^2^ = 0.022, see Figure 3C. The simple effect analysis revealed that the anticipatory emotion decreased only in the non‐social context (*p* = 0.045), but remained comparable to the pre‐training level in the social context (*p* = 0.829). In terms of the anticipatory emotion arousal in negatively forecasted events, the Condition‐by‐Time‐Point interaction was significant (*F*(1,140) = 4.334, *p* = 0.039, *partial η* ^2^ = 0.030). The simple effect analysis revealed that negative experienced emotion arousal when forecasting in non‐social context was significantly higher than in social context before WM training (*p* = 0.011), while no significant difference was found after the WM training (*p* = 0.961). Additionally, after WM training, the anticipatory negative emotion improved significantly, *F*(1,140) = 6.490, *p* = 0.012, *partial η* ^2^ = 0.044, see Figure 3D. Regarding the phenomenal characteristics of the prospected events, the details of negative events, the self‐referential details in both positive and negative events decreased significantly, *p* values ranged from 0.011 to 0.039. Notably, the Condition‐by‐Time‐Point interaction was significant in self‐referential details of positive events (*F*(1,140) = 4.461, *p* = 0.036, *partial η* ^2^ = 0.031). In non‐social context, the self‐referential details of positive events decreased significantly (*p* = 0.004), while that remained equivalent in social contexts (*p* = 0.811) (see Table S7). In the BU task, the Group‐by‐Time‐Point interaction was marginally significant in BU score for positive events (*F*(3,139) = 2.484, *p* = 0.063, *partial η* ^2^ = 0.051). Specifically, for the subgroups with SA, SD and CO, the BU scores remained unchanged after WM training, while for subgroup with CN, the BU scores marginally significantly increased (*p* = 0.058) (see Table S8). All the alteration exhibited similar in training clusters.

**FIGURE 4:**
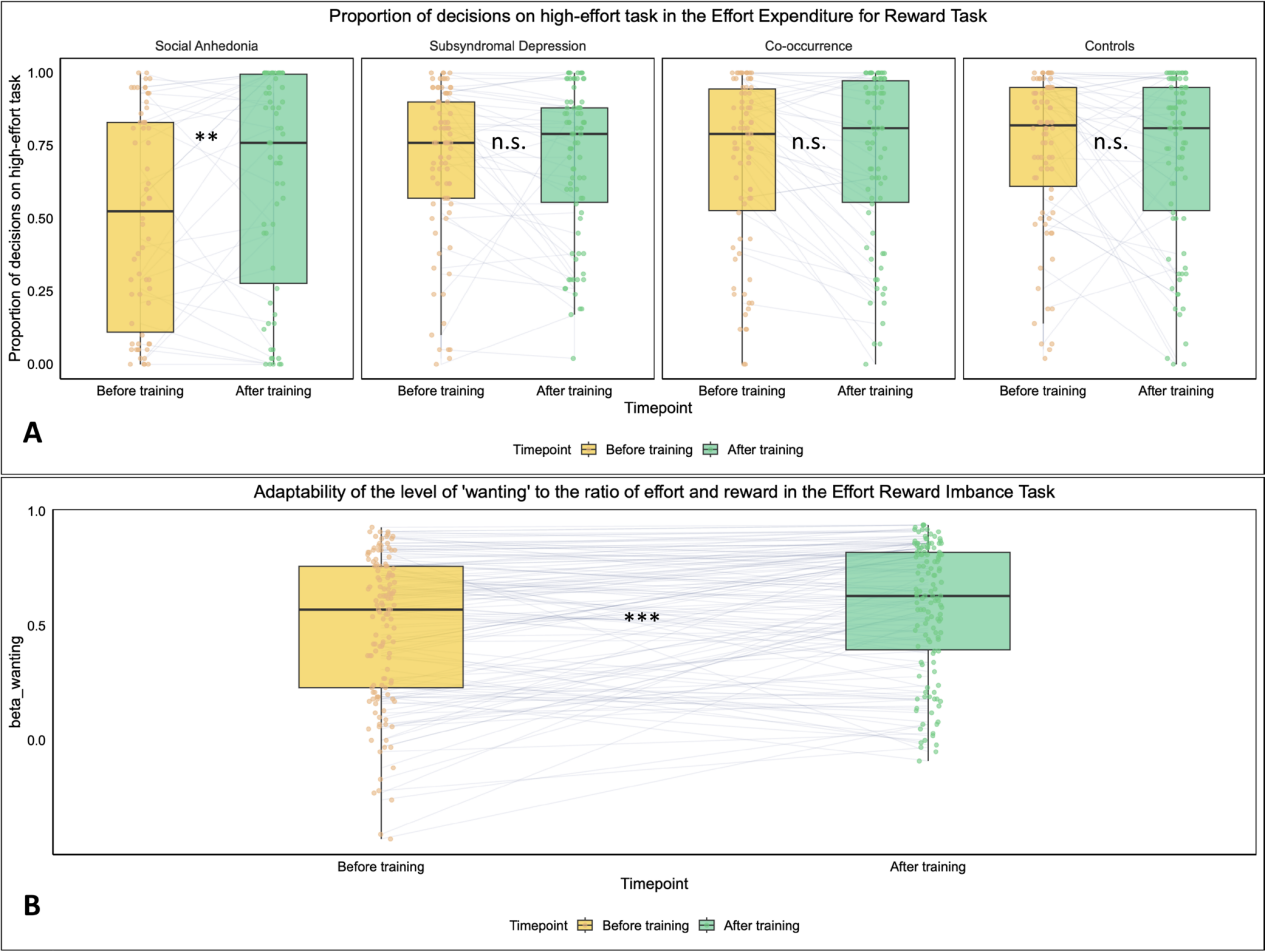
(A) Proportion of the decisions on high‐effort tasks in the Effort Expenditure for Reward Task. The individuals with high social anhedonia intend to engage more in the difficult task exclusively. (B) The adaptability of the ‘wanting’ to the ratio of effort and reward in the Effort Reward Imbalance Task. The adaptability of ‘wanting’ increased significantly after training. **p < 0.01; ***p < 0.001; n.s., not significant.

Regarding the transfer effects on adaptability of cost–benefit computation, the results did not converge. In the EEfRT task, as for the proportion of decisions on high‐effort task, the Group‐by‐Time‐Point interaction was significant, *F*(1,146) = 3.099, *p* = 0.029, *partial η* ^2^ = 0.060, see Figure 4A. The simple effect analysis exhibited that only the SA group showed increased proportion of choosing high‐effort tasks (*p* = 0.001), while the results were non‐significant for the other three groups (*p* values = 0.512–0.881). WM training enhanced the motivation to participate in difficult tasks in the group with SA. As for the self‐reported consummatory pleasure and the range adaptability of decisions and pleasure ratings with reward value, we did not observe any significant transfer effects of WM training (see Table S9). However, in the complementary clustering comparison, the interaction effect between clusters and timepoint was significant (*p* = 0.02). The consummatory pleasure increased significantly in the participants with moderate growth (*p* = 0.032) and decreased significantly in the participants with no growth (*p* = 0.033). In the ERI task, WM training increased the wanting score significantly, *F*(1,130) = 4.384, *p* = 0.038, *partial η* ^2^ = 0.033; the interaction between ratio (effort/reward) and time is significant, *F*(2,260) = 4.842, *p* = 0.019, *partial η* ^2^ = 0.036, that the increases were investigated in reward‐effort balanced and reward > effort condition. The adaptive beta for wanting increased significantly, *F*(1,130) = 13.450, *p* < 0.001, *partial η* ^2^ = 0.094, see Figure 4B, while the adaptive beta for liking increased marginally significantly, *F*(1,130) = 3.017, *p* = 0.085, *partial η* ^2^ = 0.023 (see Table S10). No significant difference was observed for the ERI in various training trajectories.

## 4 Discussion

This study comprehensively examined the effects of WM training on cognitive functions and hedonic processing in people with SA alone, people with SD alone, and people with co‐occurrence of SA and SD. We adopted a multidimensional approach to anhedonia and measured reward processing and motivation, prospection, belief updating, and cost–benefit computations before and after WM training. Overall, our findings suggested that WM training benefited WM capacity, even when different WM measures (tasks with identical versus different stimulus formats) were used. Similar benefits can be observed in executive functions. Although our primary analyses focused on participants with positive training slopes, we conducted an additional clustering analysis that included all participants to better capture the full spectrum of training patterns. The rationale behind this was that the exclusion of non‐responders with reduced engagement or atypical trajectories might have biased the interpretations of group differences. Notably, both subclinical and control groups included participants showing minimal or no improvement, suggesting that the group‐level means may have obscured the underlying heterogeneity. By clustering participants based on individual slopes, we identified five qualitatively distinct patterns of learning, providing a complementary view to the categorical group comparisons. Our findings supported Hypothesis (1) and corroborated that WM training might influence cost–benefit computation. In cost–benefit computation, the proportion of choices to engage in difficult tasks with more effort increased after training, while the pleasure was enhanced as well in the groups with moderate training growth. The results partially support Hypothesis (2). Specifically, the effectiveness of WM training appeared to be comparable between the subclinical groups and the control group as measured by most of the tasks, whilst the training resulted in increased decisions of high‐effort tasks that could be found exclusively in the individuals with high social anhedonia. Therefore, the training effects and transfer effects appeared to be generalizable across the different groups in reward processing and prospection and could alleviate avolition in people with SA. Additionally, supplementary Linear Mixed Model analyses examining both categorical groups and continuous trait scores yielded results largely consistent with our primary RM‐ANOVA findings. This convergence suggests that the observed effects are relatively robust across different statistical modeling approaches and measurement scales, although further research with more delicate designs is warranted to verify these patterns.

Previous research suggested that WM training can potentially reduce anhedonia by altering neural activation, but behavioural evidence remained limited (Li et al. 2015; Li, Li, et al. 2016). Our findings implicated the potential benefit of WM training to multiple dimensions of anhedonia, consistent with Gold et al. (2008)‘s assumption that WM is a critical cognitive underpinning for motivation and pleasure. However, such benefit may not always be found using different paradigms of measures for hedonic processing. For instance, using the BU task, we did not observe any transfer effects from WM training, given that BU may be primarily concerned with long‐term memory rather than WM (Pillny et al. 2020).

It is noteworthy that the transferred improvement for benefits computation from training was observed in people with SA, specific to this subclinical trait. After WM training, only people with SA became more willing to pay effort for the reward. Both clinical and subclinical populations of anhedonia exhibited avolition in cost–benefit computation (Wang et al. 2018). Our findings also suggested that impaired motivation in social anhedonia may be closely associated with WM deficits. Nevertheless, recent findings suggested the alteration of adaptability to reward range in SCZ patients (Wang, Gong, et al. 2024; Wang, Lui, et al. 2024). Although WM training did not improve the adaptability of engaging in high‐effort tasks, it increased the adaptability of wanting with varied ratios of effort and reward. The perception of effort‐reward imbalance was associated with high negative symptoms in SCZ patients and predicted the motivation for reward (Pan et al. 2023; Yan et al. 2021, 2023). Given that social anhedonia is a significant risk factor for SCZ, future research should employ WM training to enhance the adaptability of computations for reward and effort in SCZ patients with high negative symptoms.

The alteration after training of social and nonsocial related task contexts appeared to differ. In the SID task, which involves conditions of social punishment, we observed significantly enhanced avoidance motivation after WM training, as evidenced by the decreased anticipatory emotion. However, this effect was absent in reward or neutral targets. By contrast, the anticipatory pleasure decreased for monetary reward, as measured by the MID task. Additionally, when forecasting the future events, anticipated positive emotion for non‐social events decreased, whereas it remained stable for social events. We speculate that WM training might have a differential effect on anticipatory processing related to social and non‐social information. The reduced anticipatory positive emotions in the non‐social context may be due to participants' lower motivation to be engaged in an experiment after prolonged online training. In contrast, the corresponding stable anticipatory emotions and improved motivation in the social context could be attributable to the benefits of WM training. Previous studies have demonstrated the importance of WM for affective processing (Frank et al. 2021), providing initial support for the social benefits of WM training. However, this behavioural study did not provide further evidence to support this speculation. According to the clustering analysis, these changes are similar in all the training trajectories. The lack of cluster‐specific effects suggested that the impact of WM training might either be consistent across participants or insufficient to produce differential effects across the learning trajectories. Future studies may benefit from adjusting training intensity or using more sensitive behavioural measures to detect individual differences.

Several limitations should be acknowledged. First, this study did not account for practice effect and placebo effect for repeated administration of the tasks before and after the two‐week interval. Nonetheless, the complementary clustering analysis may address the lack of control groups by allowing comparisons across different training trajectories, minimizing the confounding influence of repeated practice. Second, we administered the tasks online, and the dropout rates were high. To address potential bias from high attrition, we compared the demographics and trait scores between completers and dropouts. The findings suggested that our final sample remains representative and that attrition was not driven by trait characteristics. Third, it remained unclear whether participants persevered during online WM training, although the clustering analysis made a complementary illustration for the training effects. In fact, the modest effect size of our WM training may be related to the online mode of administration. Further studies should recruit a larger sample adopting more robust analysis methods such as bootstrap, and conduct face‐to‐face training. Moreover, we only conducted behavioural measurements of the potential impact of WM training. Further neuropsychological evidence is needed to clarify the potential transfer effect of WM training. Furthermore, ‘affective WM’ is more closely related to social reward and affective forecasting (Frank et al. 2021), but we did not examine this important construct. Given the current evidence together supporting that the association of neural plasticity with anticipation in brain activation in both clinical and subclinical population (Li et al. 2015; Li, Li, et al. 2016), future research should investigate how WM training could alter neural activation in both clinical and subclinical samples, using the multidimensional framework of anhedonia.

In conclusion, our findings suggested that WM training could benefit reward processing and cost–benefit computation and may alleviate anhedonia. The training effects have the potential to be generalized to both subclinical and healthy individuals.

## Funding

This work was supported by the Scientific Foundation of Institute of Psychology, Chinese Academy of Sciences (E2CX3415CX) and the Philip K. H. Wong Foundation.

## Conflicts of Interest

The authors declare no conflicts of interest.

## Supporting information


**Figure S1:** Participants recruitment procedure.
**Table S1:** Comparisons of demographic characteristics and trait scores between participants who completed the training and dropouts within each group.
**Table S2:**. Participants involvement in each experimental task.
**Table S4:‐2** LMM results for cognitive gains in WM capacity.
**Table S4:‐3** LMM Results for cognitive gains in WM capacity with trait scores as continuous predictors.
**Table S5:‐2** LMM analysis of the monetary incentive delay task across groups.
**Table S5:‐3** LMM analysis of monetary incentive delay task with continuous trait scores.
**Table S6:‐2** LMM analysis of the social incentive delay task across groups.
**Table S6:‐3** LMM analysis of Social Incentive Delay Task with continuous trait scores.
**Table S7:‐2** Phenomenal characteristics of forecasted events in the social affective forecasting task.
**Table S7:‐3** LMM analysis of social affective forecasting across groups.
**Table S7:‐4** LMM analysis of social affective forecasting with continuous trait scores.
**Table S8:‐2** LMM analysis of belief updating across groups.
**Table S8:‐3** LMM analysis of belief updating with continuous trait scores.
**Table S9:‐2** LMM analysis of the EEfRT across groups.
**Table S9:‐3** LMM analysis of EEfRT with continuous trait scores.
**Table S10:‐2** LMM analysis of the ERI across groups.
**Table S10:‐3** LMM analysis of ERI with continuous trait scores.
**Table S11:‐3** LMM analysis of SART acorss groups.
**Table S11:‐4** LMM analysis of SART with continuous trait scores.

## Data Availability

The data that support the findings of this study are available on request from the corresponding author. The data are not publicly available due to privacy or ethical restrictions.
