# Supplementary material for: The Effects of Online Working Memory Training on Enhancing Hedonic Processing in People With Social Anhedonia and Subsyndromal Depression: An Exploratory Study
Source: Psych J. 2026 Feb 9;15(1):e70084. doi: 10.1002/pchj.70084 (PMC12885620; doi:10.1002/pchj.70084)
Supplement: Supplementary file 1 — Figure S1: Participants recruitment procedure. Table S1: Comparisons of demographic characteristics and trait scores between participants who completed the training and dropouts within each group. Table S2:. Participants involvement in each experimental task. Table S4:‐2 LMM results for cognitive gains in WM capacity. Table S4:‐3 LMM Results for cognitive gains in WM capacity with trait scores as continuous predictors. Table S5:‐2 LMM analysis of the monetary incentive delay task across groups. Table S5:‐3 LMM analysis of monetary incentive delay task with continuous trait scores. Table S6:‐2 LMM analysis of the social incentive delay task across groups. Table S6:‐3 LMM analysis of Social Incentive Delay Task with continuous trait scores. Table S7:‐2 Phenomenal characteristics of forecasted events in the social affective forecasting task. Table S7:‐3 LMM analysis of social affective forecasting across groups. Table S7:‐4 LMM analysis of social affective forecasting with continuous trait scores. Table S8:‐2 LMM analysis of belief updating across groups. Table S8:‐3 LMM analysis of belief updating with continuous trait scores. Table S9:‐2 LMM analysis of the EEfRT across groups. Table S9:‐3 LMM analysis of EEfRT with continuous trait scores. Table S10:‐2 LMM analysis of the ERI across groups. Table S10:‐3 LMM analysis of ERI with continuous trait scores. Table S11:‐3 LMM analysis of SART acorss groups. Table S11:‐4 LMM analysis of SART with continuous trait scores. [file PCHJ-15-e70084-s001.docx]

## Appendix A

**Supplementary Figure S1: Participants recruitment procedure**


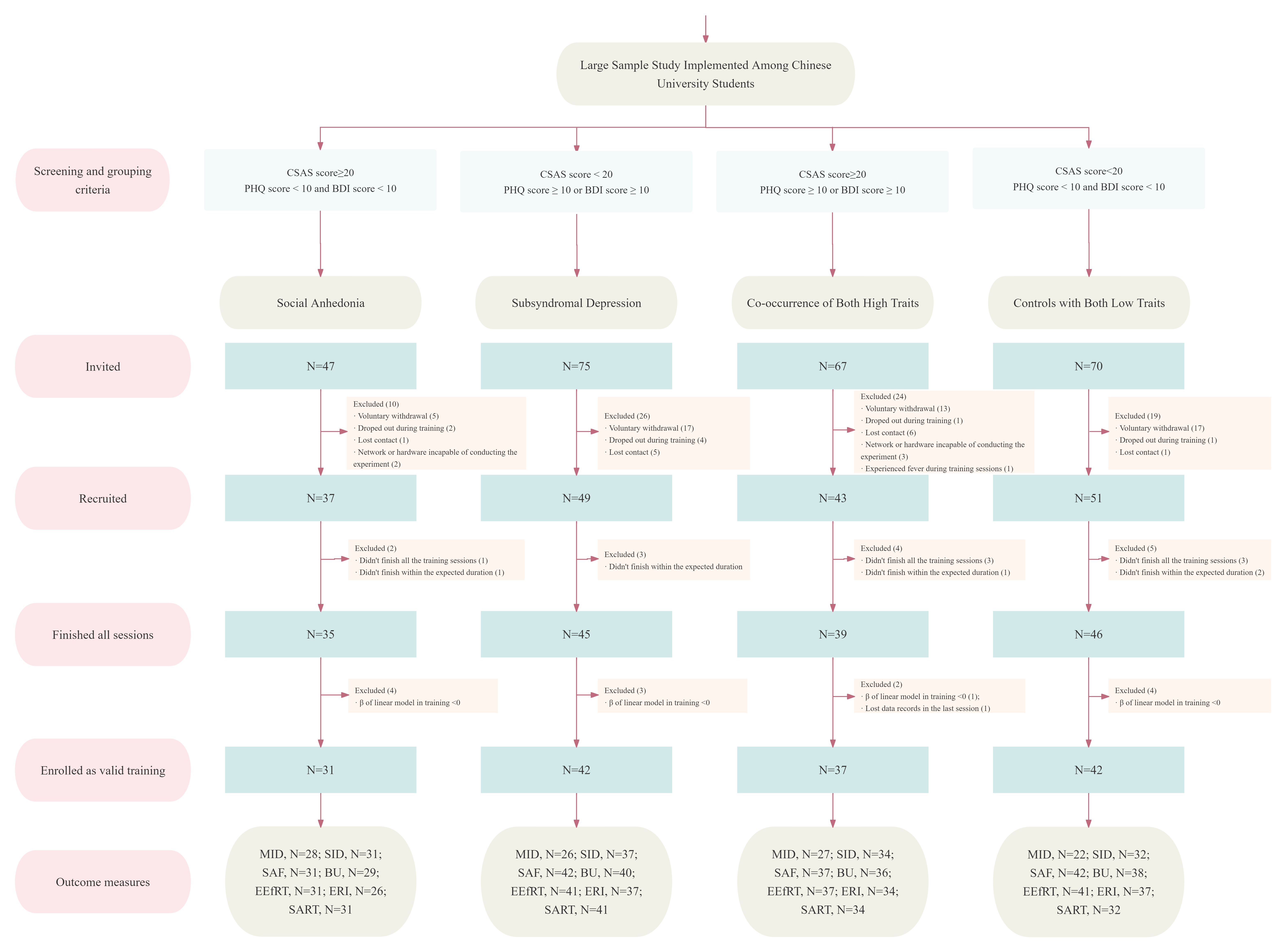


## Sample size estimation

We used G*Power 3.1.9.6 to estimate the required sample size for the present study. The effect size was determined according to the information from prior studies adopting WM training in subclinical individuals with subsyndromal depression or social anhedonia (Li et al., 2016; Zhang et al., 2019). Previous literature reported a moderate effect size f =0.30 for the session main effects and no significant interaction effects for session × group. Given that the present study aimed to investigate both session main effects and session × group interaction effects, a conservative small to moderate effect size (f = 0.15) was assumed for the interaction term. The power analysis was based on a repeated measures ANOVA design with 4 groups and 6 repeated measurements (pre/post × conditions), an alpha level of 0.05, statistical power of 0.80, an assumed correlation of 0.50 among repeated measures, and a nonsphericity correction of 0.80. The power analysis indicated that a total sample size of 104 participants was required. Based on the cut-off scores of the trait scores, we finally recruited 47 participants with SA (social anhedonia), 75 participants with SD (subsyndromal depression), 67 participants with CO (co-occurrence of social anhedonia and subsyndromal depression), and 70 participants with CN (controls).

## Attrition analysis

A chi-square test indicated that attrition rates did not differ significantly among groups (*χ²*(3) = 1.629, *p* = .653, *φ* = .079). To further examine potential attrition bias, ANOVAs were conducted within each group to compare demographic characteristics and baseline trait scores between participants who completed all training sessions and those who dropped out. Within the SA and CO groups, participants who dropped out were significantly younger than completers. No significant differences were observed in gender distribution across completers and dropouts in any group. Regarding trait measures, participants who dropped out in the CO group reported significantly higher BDI scores compared with completers, whereas PHQ scores did not differ significantly between completers and dropouts. No other significant differences were found for trait measures across groups. Overall, these results indicate that attrition was largely unrelated to baseline levels of social anhedonia or depressive symptoms, suggesting that the participants who completed the training were broadly representative of the originally recruited sample.

Table S1. Comparisons of demographic characteristics and trait scores between participants who completed the training and dropouts within each group

|  | Social Anhedonia | | | | | Subsyndromal Depression | | | | | Co-occurrence | | | | | Controls | | | | |
| --- | --- | --- | --- | --- | --- | --- | --- | --- | --- | --- | --- | --- | --- | --- | --- | --- | --- | --- | --- | --- |
|  | Trained (N=31) | Dropout (N=16) | *F*/*χ*^2^ | *p* | *η*^2^/*φ* | Trained (N=42) | Dropout (N=33) | *F*/*χ*^2^ | *p* | *η*^2^/*φ* | Trained (N=37) | Dropout (N=30) | *F*/*χ*^2^ | *p* | *η*^2^/*φ* | Trained (N=42) | Dropout (N=28) | *F*/*χ*^2^ | *p* | *η*^2^/*φ* |
| Age (year) | 20.39(1.48) | 19.31(1.20) | 6.318 | **0.016** | 0.123 | 20.62(1.71) | 21.12(1.85) | 1.483 | 0.227 | 0.020 | 20.86(2.88) | 19.67(1.63) | 4.123 | **0.046** | 0.060 | 20.45(2.02) | 19.57(1.48) | 3.936 | 0.051 | 0.055 |
| %  Male | 9.68% | 12.50% | 0.088 | 0.766 | 0.043 | 28.57% | 36.36% | 0.516 | 0.473 | 0.083 | 27.03% | 50.00% | 3.738 | 0.053 | 0.236 | 19.05% | 32.14% | 1.567 | 0.211 | 0.150 |
| BDI | 4.90(3.10) | 4.00(3.03) | 0.908 | 0.346 | 0.020 | 16.98(7.1) | 17.27(8.8) | 0.026 | 0.872 | 0.000 | 20.35(8.31) | 25.73(9.77) | 5.934 | **0.018** | 0.084 | 3.31(3.22) | 2.61(2.66) | 0.915 | 0.342 | 0.013 |
| PHQ | 4.90(2.60) | 5.31(3.14) | 0.227 | 0.636 | 0.005 | 10.86(4.36) | 12.12(4.18) | 1.610 | 0.208 | 0.022 | 13.22(5.49) | 13.27(3.94) | 0.002 | 0.966 | 0.000 | 4.19(2.7) | 4.18(2.39) | 0.000 | 0.985 | 0.000 |
| CSAS | 23.61(3.36) | 24.00(2.92) | 0.152 | 0.698 | 0.003 | 12.9(4.53) | 12.67(4.4) | 0.052 | 0.820 | 0.001 | 25.62(4.5) | 24.6(3.74) | 0.991 | 0.323 | 0.015 | 5.88(2.46) | 6.71(2.89) | 1.673 | 0.200 | 0.024 |

## Appendix B

**Procedure of the outcome measure tasks**

**The monetary incentives delay (MID) task and the social incentives delay (SID) task**

The MID/SID tasks employ 3 (reward/punishment/neutral) conditions. During the task, at the commencement of each trial, participants were presented with one of three cues (triangle, square, or circle) at the center of the screen, denoting reward, neutral, and punishment conditions, respectively, for a duration of 250ms. Subsequently, participants were required to evaluate the anticipated pleasure of the feedback on a scale from 1 to 9 based on the presented cue stimulus. Following a delay phase lasting 2000 to 2500ms, a target (a blue plus sign) was presented, and participants were instructed to respond as quickly as possible by pressing a designated key with their right index finger. The program ensured a 66% accuracy rate, with the duration of the target stimulus contingent upon individual performance on Simple RT.

Following the disappearance of the target stimulus, feedback regarding whether the participant successfully hit the target was presented for 500ms. This was followed by the feedback period, lasting 1650ms for the MID task and 3000ms for the SID task. Finally, participants were required to evaluate the pleasantness of the feedback on a scale from 1 to 9, with 1 indicating very unpleasant and 9 indicating very pleasant. Each task comprised a total of 60 trials, with 20 trials per condition.

In the MID task, monetary rewards were associated with feedback, wherein successfully hitting the target resulted in a gain of 5 points, while missing the target incurred a loss of 5 points. No monetary rewards or penalties in the neutral condition. In the SID task, reward conditions involved the presentation of a cartoon image depicting an approving facial expression upon correct target hits, while punishment conditions displayed a cartoon image depicting a disdain facial expression upon target misses; all other instances presented neutral facial expression cartoon images.

**The social affective forecasting (SAF) task**

The SAF task employs a 2x2 design, with factors of social/non-social and positive/negative valence, resulting in four conditions: positive social, positive non-social, negative social, and negative non-social. The task comprises eight events, with each condition featuring two daily events, such as 'To have a cozy family reunion dinner'. Prior to the formal experiment, an assessment exercise was conducted to ensure that participants could correctly distinguish between anticipatory and anticipated emotion. After that, participants read the material and imagined the occurrence of the presented event. They then described their forecasted events and rated the valence (1: very unhappy; 9: very happy) and arousal (1: very peaceful; 9: very excited) of their anticipatory and anticipated emotions. For positive events, they also reported the effort they would par to complete the forecast in future. Finally, participants rated the details of their imaginings: 1) sensory details including visualization, voice, smell, taste and touch (1: none; 7: many); 2) self-referential thoughts (1: none; 7: very detailed); 3) other-referential thoughts (1: none; 7: very detailed); and 4) communications (1: none; 7: very detailed). The order of presentation of the eight events was counterbalanced.

**The belief updating (BU) task**

The task consisted of 72 trials, 44 positive and 28 negative daily life events. Each trial began with a brief description of a positive or negative daily life event displayed at the center of the screen for 4 seconds. Participants were then asked to estimate the probability of the event occurring to them in the next month, using a numeric keyboard within a response window of 6 seconds. After the initial estimation, participants were informed of the base rate of the event occurring in a month for 2 seconds. The base rate for each event was the average probability of the event happening to an individual living in a similar sociocultural environment. Participants were then prompted to adjust their estimation within a 6-second response window using the numeric keyboard. Participants were informed that there were no correct or incorrect answers. Standardized instructions were provided to the participants before the experimental phase. To ensure clear understanding of the task instructions, participants completed two practice trials before the formal trials. The base rate of events was determined from the initial estimation, with a random percentage ranging from 17% to 40% being added to or subtracted from the initial estimation.

**The effort expenditure for reward task (EEfRT)**

In the EEfRT, participants were first required to decide on each trial by pressing the R key to select a high-effort task or the U key to select a low-effort task; the decision process lasted 6000 ms, and if the participant did not complete the decision, a random task was assigned to the participant. For the high-effort task, if the number of keystrokes exceeds the pass line, the participant receives a lower reward (￥5.4 to ￥6.4 tokens) or a higher reward (￥5.4 to ￥9.4 tokens), and for the low-effort task, if the number of keystrokes exceeds the pass line, the participant receives 5 tokens. The passing criteria for the high effort task was 20 consecutive keystrokes with the right index finger in 4 seconds, and for the low effort task, it was 10 consecutive keystrokes with the right index finger in 4 seconds. Once the task was completed, participants were paid the amount of that trial's reward, which they had been informed of during the decision phase of the task. The high and low prizes had an equal probability of occurring at 50% each, with a total of 80 trials per trial. The proportion of participants who chose the high-effort task across all trials reflects the strength of the motivation to pay for the reward.

**The effort reward imbalance (ERI) task**

The task employed a block design consisting of 60 trials within a 20-minute timeframe. Participants were asked to rate their desire for potential rewards using a 1-to-7 Likert scale (1 indicating no desire and 7 indicating strong desire). The participants were asked to complete six mental arithmetic items, which included randomly presented red or blue numbers. They were required to perform a 'plus eight' mental arithmetic task when presented with red numbers and a 'minus eight' task when presented with blue numbers. Afterward, they had to indicate whether the answer presented on the screen was correct or not by pressing the 'f' or 'j' key. The reward for each correct response ranged from 5 to 8 tokens. The keypress response was counterbalanced among participants. Subsequently, participants were asked to enter their expected reward value based on the accuracy of six mental arithmetic items presented and according to the reward token range. They were also required to rate their anticipated emotion on a 1-to-7 Likert scale of emotion valence (1: very unhappy; 7: very happy) if they were to receive the expected reward. Finally, participants received the actual reward. Participants were asked to compare the expected reward with the actual reward and then self-report their current emotion on a 1-to-7 Likert scale of emotion valence (1: very unhappy; 7: very happy). Objective indexes, such as mental arithmetic accuracy and reaction time of ratings, were used to control data quality. Only participants with an average accuracy of over 60% were included in the final analyses. Trials with a reaction time of ratings less than 200 ms were deemed invalid.

**The Sustained Attention to Response Task (SART)**

In the task, a total of 225 single digits (25 of each of the 9 digits) were presented visually to participants over a 4.3-minute period. Each digit was presented for 250 ms, followed by a 900-ms mask. Participants were told to respond by pressing a key to each digit, except the digit 3, when they were told to withhold a response. Participants were asked to give equal importance to accuracy and speed in performing the task. The digits were presented in 1 of 5 randomly allocated font sizes to enhance demand for processing the numerical value, rather than simply setting for a search template for some peripheral feature of the no-response target. Each session was preceded by a practice period consisting of 18 presentations of digits, 2 of which were targets.

**Appendix C**

**Quality Control and Outlier Management across Behavioral Tasks**

To ensure the integrity and reliability of the online experimental data, a rigorous multi-stage quality control protocol was implemented. First, participants’ training progress was monitored daily. A team of five trained researchers was dedicated to overseeing the training process. Each researcher was assigned a subset of participants to monitor their daily progress via the backend server. Participants were required to complete 10 training sessions within a 14-day window. Automated and manual reminders were sent if a participant missed a scheduled session. Failure to complete all 10 sessions within the designated 2-week period and Loss of contact (no response to reminders) for three consecutive days were marked as dropouts.

Second, for the pre- and post-training behavioral assessments, we applied task-specific validity criteria to identify and exclude low-quality data or outliers. (1) Physiological Plausibility: Trials with unrealistically fast reaction times (e.g., <100ms or <200ms, depending on task constraints) were excluded to minimize noise from accidental key presses. (2) Performance Accuracy: To ensure participants were engaged and understood the instructions, minimum accuracy thresholds were applied (e.g., >60% accuracy in the ERI task; ≥6 hit trials in the MID/SID tasks). Participants falling below these thresholds were excluded from the respective task analyses. (3) Data Completeness: Only participants with complete datasets for specific task components (e.g., all SAF simulations) were included. A pair-wise analysis approach was used for pre-post comparisons to maximize the inclusion of valid data points while maintaining statistical power. (See Table S2 for detailed inclusion numbers per task).

**Supplementary Linear Mixed Models (LMM) Analysis**

To complement the primary repeated measures ANOVA and further account for individual variability, we conducted LMM analyses. The models were fitted using the lme4 package with R studio. Group, condition and time point were entered as fixed effects, with random intercepts specified for each participant:

$$Y\sim Group \times Condition \times Time+\left( 1 \right| Subject)$$

Additionally, to address the potential overlap between subclinical constructs, we performed LMMs treating social anhedonia (CSAS) and depressive symptoms (BDI/PHQ) as continuous predictors.

$$Y\sim Trait Score \times Condition \times Time+\left( 1 \right| Subject)$$

The LMM results are presented in Tables S4–S11. Notably, the statistical conclusions derived from the LMMs are generally consistent with the primary RM-ANOVA findings, confirming the robustness of the reported training and transfer effects across different statistical approaches.

**Results**

**Supplementary Table S2.Participants involvement in each experimental task**

|  | Social anhedonia | Subsyndromal depression | co-occurrence | Controls |
| --- | --- | --- | --- | --- |
| **WM training** | 31 | 42 | 37 | 42 |
| **SART** | 31 | 41 | 35 | 41 |
| **MID** | 27 | 27 | 27 | 22 |
| **SID** | 31 | 38 | 34 | 32 |
| **SAF** | 30 | 38 | 36 | 40 |
| **BU** | 29 | 40 | 36 | 38 |
| **EEfRT** | 31 | 41 | 37 | 41 |
| **ERI** | 26 | 37 | 34 | 37 |

**Supplementary Table S3. The slope and intercept of the fitted liner model.**

|  | Slope_max | Intercept_max | Slope_average | Intercept_average |
| --- | --- | --- | --- | --- |
| SA (N=31) | 0.28(0.17) | 3.91(1.02) | 0.18(0.09) | 2.98(0.73) |
| SD (N=42) | 0.31(0.20) | 3.59(0.79) | 0.21(0.14) | 2.76(0.61) |
| CO (N=37) | 0.28(0.14) | 4.15(1.07) | 0.19(0.10) | 3.10(0.72) |
| CN(N=42) | 0.27(0.16) | 3.96(1.07) | 0.18(0.11) | 3.03(0.72) |
| *F_Group_* | 0.587 | 2.251 | 0.610 | 1.810 |
| *p* | 0.624 | 0.085 | 0.610 | 0.148 |
| *η*^2^*_p_* | 0.012 | 0.044 | 0.012 | 0.035 |

***Note.*** Slope_max/average, the slope of model with N-max / N-average as dependent variable ; Intercept_max/average, the intercept of model with N-max/N-average as dependent variable. SA, social anhedonia, SD, subsyndromal depression, CO, co-occurrence of social anhedonia and subsyndromal depression, CN, controls with both low trait of social anhedonia and subsyndromal depression.

**Supplementary Table S4-1. The cognitive gains on WM capacity, results from the repeated measure ANOVA.**

|  |  | N_max | N_average | LNS_longest | LNS_sum |
| --- | --- | --- | --- | --- | --- |
| SA (N=31) | pre | 1.87(0.50) | 1.44(0.25) | 6.90(1.40) | 19.19(4.47) |
|  | post | 3.48(1.12) | 2.24(0.56) | 7.74(1.41) | 22.58(5.82) |
| SD (N=42) | pre | 2.17(0.88) | 1.59(0.46) | 6.36(1.57) | 17.86(5.20) |
|  | post | 3.14(1.12) | 2.07(0.56) | 7.12(1.66) | 21.31(6.11) |
| CO (N=37) | pre | 2.00(0.71) | 1.50(0.35) | 6.92(1.50) | 18.43(5.22) |
|  | post | 3.08(1.12) | 2.04(0.56) | 7.62(1.36) | 21.68(5.29) |
| CN(N=42) | pre | 1.93(0.46) | 1.46(0.23) | 7.40(1.40) | 20.60(4.52) |
|  | post | 3.14(1.28) | 2.07(0.64) | 8.07(1.30) | 22.36(4.51) |
| *F_Group_* | | 1.443 | 1.470 | 3.901 | 1.235 |
| *p* | | 0.233 | 0.225 | 0.010 | 0.299 |
| *η*^2^*_p_* | | 0.029 | 0.029 | 0.074 | 0.025 |
| *F_Time_* | | 132.086 | 129.762 | 45.679 | 77.157 |
| *p* | | **0.000** | **0.000** | **0.000** | **0.000** |
| *η*^2^*_p_* | | 0.475 | 0.471 | 0.238 | 0.346 |
| *F_Group×Time_* | | 1.430 | 1.425 | 0.119 | 1.842 |
| *p* | | 0.236 | 0.238 | 0.949 | 0.142 |
| *η*^2^*_p_* | | 0.029 | 0.028 | 0.002 | 0.036 |

***Note.*** N_max, the maximum n of the sessions in the Dual-N-Back Task; N_average, the average of the n in all sessions; LNS_longest, the longest term successfully passed in Letter Number Span Task; LNS_sum, the total number of terms successfully passed; Pre, results before the WM training; Post, results after the WM training.

**Supplementary Table S4-2. LMM Results for Cognitive Gains in WM Capacity.**

| Metric | Source | *df_num_,df_den_* | *F* | *p* | *η^2^_p_* |
| --- | --- | --- | --- | --- | --- |
| LNS_longest | Time | 1, 149 | 43.350 | < .001*** | 0.225 |
|  | Group × Time | 3, 149 | 0.170 | 0.917 | 0.003 |
| LNS_sum | Time | 1, 149 | 75.620 | < .001*** | 0.337 |
|  | Group × Time | 3, 149 | 1.500 | 0.218 | 0.029 |
| N_max | Time | 1, 149 | 130.560 | < .001*** | 0.467 |
|  | Group × Time | 3, 149 | 1.540 | 0.206 | 0.030 |
| N_average | Time | 1, 149 | 128.342 | < .001*** | 0.463 |
|  | Group × Time | 3, 149 | 1.545 | 0.205 | 0.030 |

***Note.*** N_max, the maximum n of the sessions in the Dual-N-Back Task; N_average, the average of the n in all sessions; LNS_longest, the longest term successfully passed in Letter Number Span Task; LNS_sum, the total number of terms successfully passed; ***, *p*< .001. Following the WM training, all working memory metrics exhibited significant increases. No significant interaction between group and time was observed, suggesting that all groups demonstrated comparable improvements in working memory capacity.

**Supplementary Table S4-3. LMM Results for Cognitive Gains in WM Capacity with Trait Scores as Continuous Predictors.**

| Metrics | Source | *df_num_,df_den_* | *F* | *p* | *η^2^_p_* |
| --- | --- | --- | --- | --- | --- |
| LNS_longest | CSAS × Time | 1, 151 | 0.100 | 0.751 | 0.001 |
|  | BDI × Time | 1, 151 | 0.020 | 0.891 | <.001 |
|  | PHQ × Time | 1, 151 | 0.330 | 0.565 | 0.002 |
| LNS_sum | CSAS × Time | 1, 151 | 1.230 | 0.269 | 0.008 |
|  | BDI × Time | 1, 151 | 2.010 | 0.158 | 0.013 |
|  | PHQ × Time | 1, 151 | 1.280 | 0.260 | 0.008 |
| N_max | CSAS × Time | 1, 151 | 0.100 | 0.749 | 0.001 |
|  | BDI × Time | 1, 151 | 0.060 | 0.814 | <.001 |
|  | PHQ × Time | 1, 151 | 1.140 | 0.287 | 0.008 |
| N_average | CSAS × Time | 1, 151 | 0.100 | 0.749 | 0.001 |
|  | BDI × Time | 1, 151 | 0.050 | 0.821 | <.001 |
|  | PHQ × Time | 1, 151 | 1.150 | 0.286 | 0.008 |

***Note.*** CSAS, Revised Chapman Social Anhedonia Scale; BDI, Beck Depression Inventory; PHQ, Patient Health Questionnaire-9. All Interaction effects between subclinical traits and time were non-significant (all *p* > .05), suggesting that improvements in working memory capacity were not significantly moderated by the severity of social anhedonia or depressive symptoms. These findings indicate that the training gains on working memory were comparable across all the participants, regardless of their subclinical traits.

**Supplementary Table S5-1. Reaction time (RT), anticipatory pleasure (AP), consummatory pleasure when hit target (CPH) and consummatory feeling when missing the target (CPM) in the Monetary Incentive Delay Task.**

|  |  |  | RT | AP | CPH | CPM |
| --- | --- | --- | --- | --- | --- | --- |
| SA(N=27) | pre | Reward | 232.12(45.09) | 6.59(1.27) | 8(1.26) | 3.64(1.6) |
|  |  | Punishment | 237.56(29.4) | 3.5(0.61) | 5.62(1.08) | 2.05(0.87) |
|  |  | Neutral | 255.51(44.35) | 5.25(0.55) | 5.62(1) | 4.81(1.12) |
|  | post | Reward | 210.9(22.79) | 6.18(1.15) | 7.2(1.38) | 3.55(1.28) |
|  |  | Punishment | 218.36(23.71) | 3.66(1.37) | 5.37(1.27) | 2.29(1.24) |
|  |  | Neutral | 240.55(29.4) | 5.04(0.61) | 5.2(1.08) | 4.72(0.87) |
| SD(N=27) | pre | Reward | 222.11(31.24) | 6.58(1.32) | 7.42(1.24) | 3.59(1.3) |
|  |  | Punishment | 224.97(25.85) | 3.17(1.33) | 5.48(1.49) | 2.14(1.13) |
|  |  | Neutral | 247.48(28.39) | 5.14(0.52) | 5.24(0.94) | 4.78(0.88) |
|  | post | Reward | 202.98(28.78) | 6.53(1.28) | 7.11(1.47) | 3.46(1.52) |
|  |  | Punishment | 209.52(28.29) | 3.13(1.42) | 5.08(1.24) | 2.37(1.5) |
|  |  | Neutral | 232.69(36.05) | 4.95(0.71) | 5.15(0.74) | 4.86(1.03) |
| CO(N=27) | pre | Reward | 214.29(23.94) | 6.42(1.46) | 7.16(1.25) | 3.83(1.17) |
|  |  | Punishment | 222.47(22.45) | 3.75(1.63) | 5.49(1.3) | 2.59(1.28) |
|  |  | Neutral | 240.3(21.35) | 4.99(0.53) | 5.11(0.82) | 4.45(0.82) |
|  | post | Reward | 212.48(21.01) | 5.82(1.44) | 6.51(1.53) | 3.72(1.38) |
|  |  | Punishment | 218.1(18.58) | 3.6(1.34) | 4.57(1.23) | 2.68(1.43) |
|  |  | Neutral | 238.7(23.95) | 4.66(0.85) | 4.73(1.15) | 4.26(1.1) |
| CN(N=22) | pre | Reward | 218.63(27) | 6.72(1.12) | 7.64(1.02) | 3.73(1.27) |
|  |  | Punishment | 226.22(29.49) | 3.57(1.49) | 5.71(1.29) | 2.19(1.34) |
|  |  | Neutral | 247.2(37.04) | 5.09(0.38) | 5.27(1.01) | 4.78(0.83) |
|  | post | Reward | 210.55(33.5) | 6.34(1.45) | 7.07(1.32) | 4.02(1.88) |
|  |  | Punishment | 217.99(30.95) | 3.78(1.81) | 5.31(1.65) | 2.86(2.06) |
|  |  | Neutral | 245.15(38.22) | 5.27(0.97) | 5.27(1.4) | 4.99(1.42) |
| *F_Group_* | | | 0.934 | 0.843 | 1.852 | 0.328 |
| *p* | | | 0.427 | 0.474 | 0.143 | 0.805 |
| *η*^2^*_p_* | | | 0.028 | 0.025 | 0.053 | 0.010 |
| *F_Time_* | | | 10.920 | 4.722 | 27.681 | 0.817 |
| *p* | | | **0.001** | **0.032** | **0.000** | 0.368 |
| *η*^2^*_p_* | | | 0.099 | 0.046 | 0.219 | 0.008 |
| *F_cond_* | | | 150.594 | 174.574 | 227.916 | 247.319 |
| *p* | | | **0.000** | **0.000** | **0.000** | **0.000** |
| *η*^2^*_p_* | | | 0.603 | 0.638 | 0.697 | 0.714 |
| *F_Group×Time_* | | | 1.418 | 1.263 | 1.139 | 0.771 |
| *p* | | | 0.242 | 0.291 | 0.337 | 0.513 |
| *η*^2^*_p_* | | | 0.041 | 0.037 | 0.033 | 0.023 |
| *F_Group×cond_* | | | 0.359 | 0.963 | 0.272 | 2.223 |
| *p* | | | 0.866 | 0.423 | 0.939 | **0.052** |
| *η*^2^*_p_* | | | 0.011 | 0.028 | 0.008 | 0.063 |
| *F_Cond×Time_* | | | 1.145 | 4.286 | 5.968 | 4.345 |
| *p* | | | 0.308 | **0.026** | **0.004** | **0.014** |
| *η*^2^*_p_* | | | 0.011 | 0.041 | 0.057 | 0.042 |

***Note.*** Pre, results before the WM training; Post, results after the WM training. In the MID task, 27 participants in SA group, 27 participants in SD group, 27 participants in CO group and 22 participants in CN group completed both pre- and post- training measurement.

**Supplementary Table S5-2. LMM analysis of the Monetary Incentive Delay Task across groups.**

| Metrics | Source | *df_num_,df_den_* | *F* | *p* | *η^2^_p_* |
| --- | --- | --- | --- | --- | --- |
| RT | Time | 1, 495 | 29.090 | <.001*** | 0.056 |
|  | Group × Time | 3, 495 | 3.740 | .011* | 0.022 |
|  | Condition × Time | 2, 495 | 0.330 | 0.722 | 0.001 |
|  | Group × Condition × Time | 6, 495 | 0.100 | 0.996 | 0.001 |
| AP | Time | 1, 495 | 2.410 | 0.122 | 0.005 |
|  | Group × Time | 3, 495 | 0.740 | 0.531 | 0.004 |
|  | Condition × Time | 2, 495 | 1.240 | 0.291 | 0.005 |
|  | Group × Condition × Time | 6, 495 | 0.440 | 0.853 | 0.005 |
| CPH | Time | 1, 495 | 32.970 | <.001*** | 0.062 |
|  | Group × Time | 3, 495 | 1.170 | 0.321 | 0.007 |
|  | Condition × Time | 2, 495 | 1.910 | 0.150 | 0.008 |
|  | Group × Condition × Time | 6, 495 | 0.640 | 0.696 | 0.008 |
| CPM | Time | 1, 495 | 1.400 | 0.237 | 0.003 |
|  | Group × Time | 3, 495 | 1.310 | 0.271 | 0.008 |
|  | Condition × Time | 2, 495 | 1.520 | 0.219 | 0.006 |
|  | Group × Condition × Time | 6, 495 | 0.120 | 0.995 | 0.001 |

***Note***. ***, p<.001; *, p<.05; Significant Group × Time interactions were observed for RT (*p*=.011). Post-hoc comparisons revealed that RTs significantly decreased after training in the SA and SD groups ( p < .001), whereas no significant changes were observed in the CN and CO groups (*p* > .05).

**Supplementary Table S5-3. LMM analysis of Monetary Incentive Delay Task with continuous trait scores.**

| Metrics | Source | *df_num_,df_den_* | *F* | *p* | *η^2^_p_* |
| --- | --- | --- | --- | --- | --- |
| RT | CSAS × Time | 1, 505 | 0.841 | 0.360 | 0.002 |
|  | CSAS × Condition × Time | 2, 505 | 0.094 | 0.910 | <.001 |
|  | BDI × Time | 1, 505 | 0.083 | 0.773 | <.001 |
|  | BDI × Condition × Time | 2, 505 | 0.047 | 0.955 | <.001 |
|  | PHQ × Time | 1, 505 | 0.060 | 0.807 | <.001 |
|  | PHQ × Condition × Time | 2, 505 | 0.027 | 0.973 | <.001 |
| AP | CSAS × Time | 1, 505 | 0.476 | 0.491 | 0.001 |
|  | CSAS × Condition × Time | 2, 505 | 0.508 | 0.602 | 0.002 |
|  | BDI × Time | 1, 505 | 1.526 | 0.217 | 0.003 |
|  | BDI × Condition × Time | 2, 505 | 0.346 | 0.708 | 0.001 |
|  | PHQ × Time | 1, 505 | 0.541 | 0.462 | 0.001 |
|  | PHQ × Condition × Time | 2, 505 | 0.814 | 0.444 | 0.003 |
| CPH | CSAS × Time | 1, 505 | 0.712 | 0.399 | 0.001 |
|  | CSAS × Condition × Time | 2, 505 | 0.259 | 0.772 | 0.001 |
|  | BDI × Time | 1, 505 | 1.270 | 0.260 | 0.003 |
|  | BDI × Condition × Time | 2, 505 | 1.232 | 0.293 | 0.005 |
|  | PHQ × Time | 1, 505 | 0.007 | 0.935 | <.001 |
|  | PHQ × Condition × Time | 2, 505 | 0.938 | 0.392 | 0.004 |
| CPM | CSAS × Time | 1, 505 | 2.832 | 0.093 | 0.006 |
|  | CSAS × Condition × Time | 2, 505 | 0.310 | 0.734 | 0.001 |
|  | BDI × Time | 1, 505 | 1.477 | 0.225 | 0.003 |
|  | BDI × Condition × Time | 2, 505 | 0.012 | 0.988 | <.001 |
|  | PHQ × Time | 1, 505 | 3.859 | 0.050 | 0.008 |
|  | PHQ × Condition × Time | 2, 505 | 0.434 | 0.648 | 0.002 |

***Note***. All two-way (Trait×Time) and three-way (Trait×Condition×Time) interactions were non-significant ( *p*≥.05), indicating that the training-related changes in both cognitive speed (RT) and hedonic responses (AP, CPH, CPM) were not significantly moderated by the severity of social anhedonia or depressive symptoms. These results suggest that the transfer effects of WM training on reward and punishment processing were consistent across individuals regardless of the subclinical traits.

**Supplementary Table S6-1. Reaction time (RT), anticipatory pleasure (AP), consummatory pleasure when hit target (CPH) and consummatory feeling when missing the target (CPM) in the Social Incentive Delay Task.**

|  |  |  | RT | AP | CPH | CPM |
| --- | --- | --- | --- | --- | --- | --- |
| SA(N=31) | pre | Reward | 250.02(35.16) | 6.28(1.15) | 8.05(1.02) | 3.67(1.09) |
|  |  | Punishment | 255.73(27.07) | 4.68(1.17) | 5.85(1.40) | 2.57(1.11) |
|  |  | Neutral | 258.82(39.38) | 5.15(0.73) | 5.93(1.36) | 3.60(0.76) |
|  | post | Reward | 231.21(29.36) | 5.98(0.93) | 7.39(1.22) | 4.04(0.91) |
|  |  | Punishment | 237.07(27.22) | 4.57(1.22) | 5.54(0.88) | 3.00(1.16) |
|  |  | Neutral | 244.01(27.07) | 5.18(0.41) | 5.53(0.75) | 4.21(0.77) |
| SD(N=38) | pre | Reward | 248.4(41.76) | 6.18(1.02) | 7.68(1.25) | 3.55(1.00) |
|  |  | Punishment | 253.08(37.93) | 4.54(1.03) | 5.93(1.12) | 2.54(1.14) |
|  |  | Neutral | 256(35.59) | 5.01(0.67) | 6.01(1.00) | 3.60(0.96) |
|  | post | Reward | 221.46(25.98) | 6.27(1.00) | 7.30(1.25) | 4.05(1.16) |
|  |  | Punishment | 233.96(24.95) | 3.94(1.12) | 5.57(0.98) | 2.77(1.30) |
|  |  | Neutral | 237.5(30.51) | 5.05(0.53) | 5.75(0.92) | 4.13(1.10) |
| CO(N=34) | pre | Reward | 249.12(37.82) | 5.91(1.11) | 7.83(1.14) | 3.37(1.07) |
|  |  | Punishment | 258.2(33.42) | 4.58(0.96) | 5.54(1.29) | 2.36(1.09) |
|  |  | Neutral | 257.53(32.55) | 5.08(0.64) | 5.59(1.28) | 3.51(1.15) |
|  | post | Reward | 233.77(24.79) | 6.14(1.14) | 7.14(1.40) | 3.90(0.95) |
|  |  | Punishment | 240.93(21.72) | 4.05(1.00) | 4.94(0.91) | 2.70(1.20) |
|  |  | Neutral | 244.65(29.46) | 4.89(0.35) | 5.03(0.78) | 3.94(0.88) |
| CN(N=32) | pre | Reward | 246.78(32.76) | 6.14(1.06) | 7.73(0.98) | 3.97(1.35) |
|  |  | Punishment | 249.43(36.16) | 4.59(1.24) | 5.71(1.28) | 2.89(1.54) |
|  |  | Neutral | 256.8(37.98) | 5.19(0.86) | 5.89(1.27) | 4.09(1.15) |
|  | post | Reward | 227.79(32.77) | 5.99(1.19) | 7.14(1.13) | 4.16(0.70) |
|  |  | Punishment | 242.64(37.43) | 4.43(1.27) | 5.49(1.01) | 2.92(1.06) |
|  |  | Neutral | 243.61(36.56) | 5.11(0.78) | 5.56(0.98) | 4.18(0.72) |
| *F_Group_* | | | 0.334 | 0.990 | 1.497 | 1.464 |
| *p* | | | 0.800 | 0.400 | 0.218 | 0.227 |
| *η*^2^*_p_* | | | 0.008 | 0.022 | 0.033 | 0.032 |
| *F_Time_* | | | 33.072 | 7.573 | 38.442 | 16.451 |
| *p* | | | **0.000** | **0.007** | **0.000** | **0.000** |
| *η*^2^*_p_* | | | 0.202 | 0.055 | 0.227 | 0.112 |
| *F_cond_* | | | 29.629 | 143.585 | 342.095 | 223.943 |
| *p* | | | **0.000** | **0.000** | **0.000** | **0.000** |
| *η*^2^*_p_* | | | 0.184 | 0.523 | 0.724 | 0.631 |
| *F_Group×Time_* | | | 0.412 | 0.034 | 0.747 | 0.890 |
| *p* | | | 0.744 | 0.992 | 0.526 | 0.448 |
| *η*^2^*_p_* | | | 0.009 | 0.001 | 0.017 | 0.020 |
| *F_Group×cond_* | | | 0.219 | 0.617 | 1.457 | 0.085 |
| *p* | | | 0.971 | 0.630 | 0.225 | 0.998 |
| *η*^2^*_p_* | | | 0.005 | 0.014 | 0.032 | 0.002 |
| *F_Cond×Time_* | | | 1.984 | 4.948 | 3.219 | 2.158 |
| *p* | | | 0.140 | **0.016** | **0.061** | 0.119 |
| *η*^2^*_p_* | | | 0.015 | 0.036 | 0.024 | 0.016 |

***Note.*** Pre, results before the WM training; Post, results after the WM training. In the SID task, 31 participants in SA group, 38 participants in SD group, 34 participants in CO group and 32 participants in CN group completed both pre- and post- training measurement.

**Supplementary Table S6-2. LMM analysis of the Social Incentive Delay Task across groups.**

| Metrics | Source | *df_num_,df_den_* | *F* | *p* | *η^2^_p_* |
| --- | --- | --- | --- | --- | --- |
| RT | Time | 1, 655 | 99.059 | <.001*** | 0.131 |
|  | Group × Time | 3, 655 | 1.235 | 0.296 | 0.006 |
|  | Condition × Time | 2, 655 | 0.938 | 0.392 | 0.003 |
|  | Group × Condition × Time | 6, 655 | 0.360 | 0.904 | 0.003 |
| AP | Time | 1, 655 | 5.190 | 0.023* | 0.008 |
|  | Group × Time | 3, 655 | 0.023 | 0.995 | 0.000 |
|  | Condition × Time | 2, 655 | 2.523 | 0.081 | 0.008 |
|  | Group × Condition × Time | 6, 655 | 1.301 | 0.254 | 0.012 |
| CPH | Time | 1, 655 | 57.476 | <.001*** | 0.081 |
|  | Group × Time | 3, 655 | 1.114 | 0.343 | 0.005 |
|  | Condition × Time | 2, 655 | 1.265 | 0.283 | 0.004 |
|  | Group × Condition × Time | 6, 655 | 0.195 | 0.978 | 0.002 |
| CPM | Time | 1, 655 | 41.410 | <.001*** | 0.059 |
|  | Group × Time | 3, 655 | 2.245 | 0.082 | 0.010 |
|  | Condition × Time | 2, 655 | 0.806 | 0.447 | 0.002 |
|  | Group × Condition × Time | 6, 655 | 0.262 | 0.954 | 0.002 |

***Note***. ***, *p*<.001; *, *p*<.05; Significant main effects of Time were observed across all metrics (*p* < .05). Specifically, post-training assessments revealed significant decreases in RT (*p* < .001), AP (*p* = .023), and CPH (*p* < .001), alongside a significant increase in CPM (*p* < .001). No significant Interaction effects (Group × Time or Group × Condition × Time) were detected, indicating that these training-induced shifts in social reward processing were consistent across all groups.

**Supplementary Table S6-3. LMM analysis of Social Incentive Delay Task with continuous trait scores.**

| Metrics | Source | *df_num_,df_den_* | *F* | *p* | *η^2^_p_* |
| --- | --- | --- | --- | --- | --- |
| RT | CSAS × Time | 1, 665 | 0.852 | 0.356 | 0.001 |
|  | CSAS × Condition × Time | 2, 665 | 0.859 | 0.424 | 0.003 |
|  | BDI × Time | 1, 665 | 3.235 | 0.073 | 0.005 |
|  | BDI × Condition × Time | 2, 665 | 0.081 | 0.922 | 0.000 |
|  | PHQ × Time | 1, 665 | 1.687 | 0.194 | 0.003 |
|  | PHQ × Condition × Time | 2, 665 | 0.050 | 0.951 | 0.000 |
| AP | CSAS × Time | 1, 665 | 0.001 | 0.971 | 0.000 |
|  | CSAS × Condition × Time | 2, 665 | 0.223 | 0.800 | 0.001 |
|  | BDI × Time | 1, 665 | 0.244 | 0.621 | 0.000 |
|  | BDI × Condition × Time | 2, 665 | 5.085 | 0.006** | 0.015 |
|  | PHQ × Time | 1, 665 | 0.018 | 0.892 | 0.000 |
|  | PHQ × Condition × Time | 2, 665 | 5.379 | 0.005** | 0.016 |
| CPH | CSAS × Time | 1, 665 | 0.673 | 0.412 | 0.001 |
|  | CSAS × Condition × Time | 2, 665 | 0.010 | 0.990 | 0.000 |
|  | BDI × Time | 1, 665 | 0.110 | 0.740 | 0.000 |
|  | BDI × Condition × Time | 2, 665 | 0.102 | 0.903 | 0.000 |
|  | PHQ × Time | 1, 665 | 0.252 | 0.616 | 0.000 |
|  | PHQ × Condition × Time | 2, 665 | 0.486 | 0.615 | 0.001 |
| CPM | CSAS × Time | 1, 665 | 4.809 | 0.029* | 0.007 |
|  | CSAS × Condition × Time | 2, 665 | 0.038 | 0.963 | 0.000 |
|  | BDI × Time | 1, 665 | 0.480 | 0.489 | 0.001 |
|  | BDI × Condition × Time | 2, 665 | 0.105 | 0.900 | 0.000 |
|  | PHQ × Time | 1, 665 | 2.234 | 0.135 | 0.003 |
|  | PHQ × Condition × Time | 2, 665 | 0.163 | 0.850 | 0.000 |

***Note***. **, *p*<.01; Significant three-way interactions (Trait×Condition×Time) were observed for AP when moderated by BDI (*p* = .006) and PHQ (*p* = .005). Follow-up simple slope analysis revealed that these interactions were primarily driven by the Punishment condition, where individuals with moderate-to-high depressive symptoms exhibited significant decreases in AP following WM training (*p* ≤ .001); no such effects were found in Neutral or Reward conditions. Additionally, a significant CSAS × Time interaction for CPM (*p* = .029) was observed; while all participants showed increased CPM post-training, the improvement was more pronounced in individuals with higher social anhedonia.

**Supplementary Table S7-1. Anticipated, anticipatory emotion and motivation in the social affective forecasting task.**

|  |  | |  | | Anticipated emotion | | | | Anticipatory emotion | | | | Motivation |
| --- | --- | --- | --- | --- | --- | --- | --- | --- | --- | --- | --- | --- | --- |
|  |  | |  | | p-ev | n-ev | p-ea | n-ea | p-ev | n-ev | p-ea | n-ea | expected effort |
| SA (N=30) | | pre | | NS | 7.68(0.88) | 2.58(1.64) | 6.8(1.18) | 6.15(1.77) | 7.1(1.21) | 3.6(1.32) | 6.23(1.25) | 5.83(1.32) | 7.57(0.94) |
|  |  |  |  | S | 7.55(1.03) | 2.8(1.27) | 6.88(1.28) | 5.68(1.66) | 6.57(1.33) | 3.27(0.97) | 6.42(1.35) | 5.37(1.13) | 6.98(1.64) |
|  |  | post | | NS | 7.58(1.11) | 3.03(1.7) | 6.68(1.05) | 5.88(1.26) | 6.77(1.16) | 3.78(1.06) | 6.15(1.44) | 5.58(1.08) | 7.55(0.88) |
|  |  |  |  | S | 7.52(1.08) | 3.05(1.2) | 6.57(1.13) | 5.67(1.35) | 6.7(1.23) | 3.93(0.96) | 6.25(1.25) | 5.5(1.23) | 7.26(1.32) |
| SD (N=38) | | pre | | NS | 7.66(0.87) | 2.39(1.2) | 7.01(1.03) | 5.86(1.97) | 6.87(1.29) | 3.14(1.32) | 6.39(1.19) | 5.51(1.5) | 7.3(0.93) |
|  |  |  |  | S | 7.75(1.19) | 2.74(1.21) | 7.26(1.08) | 5.64(1.74) | 6.88(1.4) | 3.42(1.15) | 6.57(1.26) | 5.14(1.39) | 7.53(1.01) |
|  |  | post | | NS | 7.66(1.12) | 2.41(1.13) | 7.03(1.22) | 5.93(1.91) | 6.78(1.21) | 3.42(1.27) | 6.43(1.34) | 5.45(1.43) | 7.33(0.98) |
|  |  |  |  | S | 7.8(1.1) | 2.49(1.24) | 7.2(1.06) | 5.49(1.67) | 6.62(1.14) | 3.3(0.97) | 6.41(1.22) | 5.45(1.62) | 7.3(1.19) |
| CO (N=36) | | pre | | NS | 7.82(0.95) | 2.42(1.25) | 6.92(1.19) | 5.61(1.94) | 6.94(1.24) | 3.43(1.11) | 6.22(1.16) | 5.46(1.28) | 7.47(0.92) |
|  |  |  |  | S | 7.31(1.19) | 2.56(1.15) | 6.86(1.17) | 6.13(1.34) | 6.4(1.38) | 3.56(1.31) | 6.13(1.39) | 5.49(1.29) | 7.1(1.51) |
|  |  | post | | NS | 7.26(1.03) | 2.39(1.06) | 6.56(1.27) | 5.75(1.98) | 6.5(1.11) | 3.79(1.08) | 6.07(1.2) | 5.26(1.33) | 7.13(1.08) |
|  |  |  |  | S | 7.25(1.14) | 2.6(1.18) | 6.64(1.15) | 5.85(1.83) | 6.58(1.28) | 3.78(1.23) | 5.88(1.31) | 5.31(1.2) | 7.11(1.4) |
| CN(N=40) | | pre | | NS | 7.51(0.94) | 2.14(0.87) | 6.81(1.13) | 6.23(1.83) | 6.83(0.93) | 3.44(1.06) | 6.26(1.14) | 5.81(1.54) | 7.5(1.09) |
|  |  |  |  | S | 7.59(0.93) | 2.69(1.34) | 7.09(1.02) | 6.04(1.42) | 6.84(1.22) | 3.81(1.16) | 6.4(1.16) | 5.46(1.29) | 7.43(1.1) |
|  |  | post | | NS | 7.58(0.94) | 2.44(1.17) | 7.04(0.97) | 6.04(1.74) | 6.98(1.03) | 3.66(1.25) | 6.56(1.08) | 5.58(1.14) | 7.69(1.15) |
|  |  |  |  | S | 7.55(1.04) | 2.76(1.14) | 7.06(1.1) | 6.13(1.3) | 6.86(0.98) | 3.75(1.19) | 6.53(0.99) | 5.6(0.99) | 7.5(1.05) |
| *F_Group_* | | | | | 0.958 | 1.094 | 1.792 | 0.610 | 0.483 | 1.406 | 1.156 | 0.572 | 0.860 |
| *p* | | | | | 0.415 | 0.354 | 0.152 | 0.610 | 0.695 | 0.244 | 0.329 | 0.634 | 0.464 |
| *η*^2^*_p_* | | | | | 0.020 | 0.023 | 0.037 | 0.013 | 0.010 | 0.029 | 0.024 | 0.012 | 0.018 |
| *F_Time_* | | | | | 1.485 | 1.706 | 2.082 | 0.288 | 1.420 | 6.490 | 0.323 | 0.166 | <.001 |
| *p* | | | | | 0.225 | 0.194 | 0.151 | 0.592 | 0.235 | **0.012** | 0.571 | 0.684 | 0.992 |
| *η*^2^*_p_* | | | | | 0.010 | 0.012 | 0.015 | 0.002 | 0.010 | 0.044 | 0.002 | 0.001 | <.001 |
| *F_events_* | | | | | 0.521 | 12.218 | 1.435 | 1.280 | 4.999 | 0.773 | 0.162 | 4.122 | 2.770 |
| *p* | | | | | 0.472 | **0.001** | 0.233 | 0.260 | **0.027** | 0.381 | 0.688 | **0.044** | 0.098 |
| *η*^2^*_p_* | | | | | 0.004 | 0.080 | 0.010 | 0.009 | 0.034 | 0.005 | 0.001 | 0.029 | 0.020 |
| *F_Group×Time_* | | | | | 1.306 | 1.516 | 1.461 | 0.025 | 0.817 | 0.930 | 1.497 | 0.346 | 1.667 |
| *p* | | | | | 0.275 | 0.213 | 0.228 | 0.995 | 0.487 | 0.428 | 0.218 | 0.792 | 0.117 |
| *η*^2^*_p_* | | | | | 0.027 | 0.031 | 0.030 | 0.001 | 0.017 | 0.020 | 0.031 | 0.007 | 0.035 |
| *F_Group×Events_* | | | | | 1.215 | 1.147 | 0.521 | 2.718 | 0.675 | 0.702 | 0.683 | 0.780 | 1.161 |
| *p* | | | | | 0.306 | 0.333 | 0.669 | **0.047** | 0.569 | 0.552 | 0.564 | 0.507 | 0.327 |
| *η*^2^*_p_* | | | | | 0.025 | 0.024 | 0.011 | 0.055 | 0.014 | 0.015 | 0.014 | 0.016 | 0.025 |
| *F_Events×Time_* | | | | | 1.668 | 1.346 | 0.699 | 0.041 | 3.089 | 0.420 | 1.567 | 4.334 | 0.599 |
| *p* | | | | | 0.199 | 0.248 | 0.404 | 0.841 | **0.081** | 0.518 | 0.213 | **0.039** | 0.440 |
| *η*^2^*_p_* | | | | | 0.012 | 0.010 | 0.005 | <.001 | 0.022 | 0.003 | 0.011 | 0.030 | 0.004 |

***Note.*** Ev, emotional valence; ea, emotional arousal; p, positive events; n, negative, events; NS, non-social contexts; S, social contexts; Pre, results before the WM training; Post, results after the WM training. 30 participants in SA group, 38 participants in SD group, 36 participants in CO group and 40 participants in CN group completed both pre- and post- measurement of the SAF task.

**Supplementary Table S7-2. Phenomenal characteristics of forecasted events in the Social Affective Forecasting Task.**

|  |  | | |  | Sensory | | Event | | Self | | Others | | Communication | |
| --- | --- | --- | --- | --- | --- | --- | --- | --- | --- | --- | --- | --- | --- | --- |
|  |  | | |  | p | n | p | n | p | n | p | n | p | n |
| SA (N=30) | | pre | NS | | 4.26(1.55) | 4.26(1.14) | 5.37(1.61) | 5.39(1.55) | 6.06(1.6) | 6.31(1.41) | 3.89(1.8) | 4.48(1.92) | 3.93(2.18) | 3.97(1.76) |
|  |  |  | S | | 5.31(1.51) | 4.17(1.25) | 5.83(1.54) | 5.16(1.52) | 5.7(1.47) | 6.06(1.62) | 5.32(1.51) | 5.11(1.77) | 4.64(1.99) | 5.13(1.97) |
|  |  | post | NS | | 4.35(1.46) | 4.43(1.42) | 5.23(1.86) | 5.42(1.8) | 5.96(1.74) | 6.09(1.74) | 4.23(1.89) | 4.55(1.85) | 4.03(1.82) | 4.46(1.99) |
|  |  |  | S | | 5.55(1.59) | 4.34(1.4) | 6(1.54) | 4.94(2.01) | 5.92(1.7) | 5.95(1.69) | 5.48(1.76) | 5.36(1.89) | 5.29(1.76) | 5.39(2) |
| SD (N=38) | | pre | NS | | 4.28(1.29) | 4.36(1.42) | 4.85(2.06) | 5.62(1.62) | 6.51(1.46) | 6.1(1.71) | 3.84(2.03) | 4.43(2.16) | 3.85(1.8) | 4.16(2.05) |
|  |  |  | S | | 5.7(1.39) | 4.2(1.44) | 6.03(1.62) | 5.45(1.86) | 6.15(1.37) | 6.48(1.32) | 5.73(1.75) | 5.55(1.82) | 5.53(1.55) | 5.49(1.58) |
|  |  | post | NS | | 4.34(1.42) | 4.5(1.37) | 5.1(1.92) | 5.5(1.65) | 5.95(1.74) | 6.14(1.82) | 3.59(1.93) | 3.9(1.92) | 3.75(2.13) | 3.99(1.82) |
|  |  |  | S | | 5.46(1.59) | 4.42(1.2) | 5.94(1.7) | 5.06(1.57) | 5.98(1.54) | 6.08(1.65) | 5.25(1.83) | 4.97(1.81) | 5.45(1.94) | 4.96(1.65) |
| CO (N=36) | | pre | NS | | 4.22(1.19) | 4.29(1.21) | 5.25(2.02) | 5.73(1.73) | 5.81(1.77) | 6.33(1.6) | 3.43(2.03) | 3.97(1.94) | 3.56(2.14) | 3.81(2.05) |
|  |  |  | S | | 5.61(1.61) | 4.22(1.31) | 6.31(1.57) | 5.2(1.87) | 6.14(1.87) | 6.01(1.73) | 5.72(2.07) | 4.62(1.68) | 5.43(1.96) | 4.51(2.2) |
|  |  | post | NS | | 4(1.38) | 4.19(1.23) | 4.88(1.96) | 5.07(1.92) | 5.44(1.95) | 5.73(1.84) | 3.46(2.01) | 3.9(2.03) | 3.59(2.21) | 3.86(1.95) |
|  |  |  | S | | 5.51(1.85) | 4.21(1.39) | 5.7(1.68) | 4.77(1.78) | 6.13(1.88) | 5.76(1.84) | 5.4(1.89) | 4.68(2.06) | 5.21(2.29) | 4.61(2.11) |
| CN(N=40) | | pre | NS | | 4.31(1.52) | 4.41(1.43) | 5.25(2.06) | 5.58(1.79) | 6.49(1.7) | 6.38(1.7) | 4.11(2.32) | 4.64(2.27) | 4.31(2.45) | 4.34(2.28) |
|  |  | | | S | 5.6(1.6) | 4.39(1.29) | 6.23(1.57) | 5.32(2.14) | 6.29(1.6) | 6.63(1.7) | 5.66(1.74) | 5.21(2.15) | 5.49(1.89) | 5.34(2.01) |
|  | post | | | NS | 4.19(1.63) | 4.4(1.34) | 5.39(2.01) | 5.73(1.75) | 6.1(1.79) | 6.43(1.65) | 3.61(2.09) | 4.52(1.87) | 3.69(1.93) | 4.42(2.03) |
|  |  |  |  | S | 5.6(1.49) | 4.23(1.44) | 6.07(1.46) | 5.12(1.94) | 6.14(1.83) | 6.08(1.59) | 5.81(1.63) | 5.31(1.7) | 5.38(1.73) | 5.16(1.79) |
| *F_Group_* | | | | | 0.055 | 0.117 | 0.221 | 0.235 | 0.583 | 0.558 | 0.275 | 1.128 | 0.233 | 1.036 |
| *p* | | | | | 0.983 | 0.950 | 0.882 | 0.872 | 0.627 | 0.644 | 0.843 | 0.340 | 0.873 | 0.379 |
| *η*^2^*_p_* | | | | | 0.001 | 0.002 | 0.005 | 0.005 | 0.012 | 0.012 | 0.006 | 0.024 | 0.005 | 0.022 |
| *F_Time_* | | | | | 0.297 | 0.424 | 0.783 | 4.878 | 4.355 | 6.717 | 1.090 | 0.667 | 0.141 | 0.012 |
| *p* | | | | | 0.587 | 0.516 | 0.378 | **0.029** | **0.039** | **0.011** | 0.298 | 0.416 | 0.708 | 0.912 |
| *η*^2^*_p_* | | | | | 0.002 | 0.003 | 0.006 | 0.034 | 0.030 | 0.046 | 0.008 | 0.005 | 0.001 | <.001 |
| *F_events_* | | | | | 227.637 | 2.095 | 68.127 | 23.361 | 0.032 | 0.673 | 162.053 | 68.493 | 130.295 | 86.033 |
| *p* | | | | | **0.000** | 0.150 | **0.000** | **0.000** | 0.858 | 0.413 | **0.000** | **0.000** | **0.000** | **0.000** |
| *η*^2^*_p_* | | | | | 0.619 | 0.015 | 0.327 | 0.143 | <.001 | 0.005 | 0.537 | 0.329 | 0.482 | 0.381 |
| *F_Group×Time_* | | | | | 0.808 | 0.856 | 1.311 | 1.254 | 0.902 | 0.373 | 1.420 | 1.553 | 1.716 | 1.728 |
| *p* | | | | | 0.492 | 0.466 | 0.273 | 0.293 | 0.442 | 0.773 | 0.240 | 0.204 | 0.166 | 0.164 |
| *η*^2^*_p_* | | | | | 0.017 | 0.018 | 0.027 | 0.026 | 0.019 | 0.008 | 0.030 | 0.032 | 0.035 | 0.036 |
| *F_Group×Events_* | | | | | 0.603 | 0.156 | 0.644 | 0.157 | 3.750 | 1.272 | 1.238 | 1.090 | 1.628 | 0.841 |
| *p* | | | | | 0.614 | 0.925 | 0.588 | 0.925 | **0.013** | 0.286 | 0.298 | 0.356 | 0.186 | 0.474 |
| *η*^2^*_p_* | | | | | 0.013 | 0.003 | 0.014 | 0.003 | 0.074 | 0.027 | 0.026 | 0.023 | 0.034 | 0.018 |
| *F_Events×Time_* | | | | | 0.029 | 0.005 | 0.900 | 1.343 | 4.461 | 1.059 | 0.031 | 0.495 | 1.403 | 1.027 |
| *p* | | | | | 0.865 | 0.945 | 0.344 | 0.248 | **0.036** | 0.305 | 0.862 | 0.483 | 0.238 | 0.313 |
| *η*^2^*_p_* | | | | | <.001 | <.001 | 0.006 | 0.010 | 0.031 | 0.008 | <.001 | 0.004 | 0.010 | 0.007 |

***Note.*** P, positive events; n, negative, events; NS, non-social contexts; S, social contexts; Pre, results before the WM training; Post, results after the WM training.

**Supplementary Table S7-3. LMM analysis of social affective forecasting across groups.**

| Metrics | | Source | *df_num_,df_den_* | *F* | *p* | *η^2^_p_* |
| --- | --- | --- | --- | --- | --- | --- |
| Anticipated emotion | p-ev | Time | 1, 420 | 1.599 | 0.207 | 0.004 |
|  |  | Group × Time | 3, 420 | 1.406 | 0.240 | 0.010 |
|  |  | Events × Time | 1, 420 | 0.970 | 0.325 | 0.002 |
|  |  | Group × Events × Time | 3, 420 | 0.996 | 0.395 | 0.007 |
|  | n-ev | Time | 1, 420 | 2.182 | 0.140 | 0.005 |
|  |  | Group × Time | 3, 420 | 1.939 | 0.123 | 0.014 |
|  |  | Events × Time | 1, 420 | 1.151 | 0.284 | 0.003 |
|  |  | Group × Events × Time | 3, 420 | 0.283 | 0.838 | 0.002 |
|  | p-ea | Time | 1, 420 | 2.419 | 0.121 | 0.006 |
|  |  | Group × Time | 3, 420 | 1.697 | 0.167 | 0.012 |
|  |  | Events × Time | 1, 420 | 0.487 | 0.486 | 0.001 |
|  |  | Group × Events × Time | 3, 420 | 0.392 | 0.759 | 0.003 |
|  | n-ea | Time | 1, 420 | 0.493 | 0.483 | 0.001 |
|  |  | Group × Time | 3, 420 | 0.042 | 0.989 | 0.000 |
|  |  | Events × Time | 1, 420 | 0.023 | 0.881 | 0.000 |
|  |  | Group × Events × Time | 3, 420 | 0.669 | 0.572 | 0.005 |
| Anticipatory emotion | p-ev | Time | 1, 420 | 1.488 | 0.223 | 0.004 |
|  |  | Group × Time | 3, 420 | 0.856 | 0.464 | 0.006 |
|  |  | Events × Time | 1, 420 | 2.270 | 0.133 | 0.005 |
|  |  | Group × Events × Time | 3, 420 | 2.418 | 0.066 | 0.017 |
|  | n-ev | Time | 1, 420 | 8.136 | 0.005** | 0.019 |
|  |  | Group × Time | 3, 420 | 1.165 | 0.323 | 0.008 |
|  |  | Events × Time | 1, 420 | 0.302 | 0.583 | 0.001 |
|  |  | Group × Events × Time | 3, 420 | 1.479 | 0.220 | 0.010 |
|  | p-ea | Time | 1, 420 | 0.395 | 0.530 | 0.001 |
|  |  | Group × Time | 3, 420 | 1.832 | 0.141 | 0.013 |
|  |  | Events × Time | 1, 420 | 1.007 | 0.316 | 0.002 |
|  |  | Group × Events × Time | 3, 420 | 0.041 | 0.989 | 0.000 |
|  | n-ea | Time | 1, 420 | 0.271 | 0.603 | 0.001 |
|  |  | Group × Time | 3, 420 | 0.565 | 0.638 | 0.004 |
|  |  | Events × Time | 1, 420 | 2.801 | 0.095 | 0.007 |
|  |  | Group × Events × Time | 3, 420 | 0.284 | 0.837 | 0.002 |
| Motivation | expected effort | Time | 1, 420 | 0.583 | 0.446 | 0.001 |
|  |  | Group × Time | 3, 420 | 1.061 | 0.365 | 0.008 |
|  |  | Events × Time | 1, 420 | 0.087 | 0.768 | 0.000 |
|  |  | Group × Events × Time | 3, 420 | 1.092 | 0.352 | 0.008 |
| Sensory | p | Time | 1, 420 | 0.297 | 0.586 | 0.001 |
|  |  | Group × Time | 3, 420 | 0.809 | 0.490 | 0.006 |
|  |  | Events × Time | 1, 420 | 0.018 | 0.894 | 0.000 |
|  |  | Group × Events × Time | 3, 420 | 0.557 | 0.644 | 0.004 |
|  | n | Time | 1, 420 | 0.672 | 0.413 | 0.002 |
|  |  | Group × Time | 3, 420 | 1.356 | 0.256 | 0.010 |
|  |  | Events × Time | 1, 420 | 0.003 | 0.959 | 0.000 |
|  |  | Group × Events × Time | 3, 420 | 0.214 | 0.886 | 0.002 |
| Event | p | Time | 1, 420 | 5.042 | 0.025* | 0.012 |
|  |  | Group × Time | 3, 420 | 1.044 | 0.373 | 0.007 |
|  |  | Events × Time | 1, 420 | 3.665 | 0.056 | 0.009 |
|  |  | Group × Events × Time | 3, 420 | 0.030 | 0.993 | 0.000 |
|  | n | Time | 1, 420 | 9.999 | 0.002** | 0.023 |
|  |  | Group × Time | 3, 420 | 0.555 | 0.645 | 0.004 |
|  |  | Events × Time | 1, 420 | 0.790 | 0.375 | 0.002 |
|  |  | Group × Events × Time | 3, 420 | 2.008 | 0.112 | 0.014 |
| Self | p | Time | 1, 420 | 1.079 | 0.300 | 0.003 |
|  |  | Group × Time | 3, 420 | 1.807 | 0.145 | 0.013 |
|  |  | Events × Time | 1, 420 | 0.477 | 0.490 | 0.001 |
|  |  | Group × Events × Time | 3, 420 | 0.526 | 0.664 | 0.004 |
|  | n | Time | 1, 420 | 7.255 | 0.007** | 0.017 |
|  |  | Group × Time | 3, 420 | 1.865 | 0.135 | 0.013 |
|  |  | Events × Time | 1, 420 | 0.893 | 0.345 | 0.002 |
|  |  | Group × Events × Time | 3, 420 | 0.602 | 0.614 | 0.004 |
| Others | p | Time | 1, 420 | 0.946 | 0.331 | 0.002 |
|  |  | Group × Time | 3, 420 | 1.232 | 0.298 | 0.009 |
|  |  | Events × Time | 1, 420 | 0.017 | 0.895 | 0.000 |
|  |  | Group × Events × Time | 3, 420 | 1.132 | 0.336 | 0.008 |
|  | n | Time | 1, 420 | 0.975 | 0.324 | 0.002 |
|  |  | Group × Time | 3, 420 | 2.272 | 0.080 | 0.016 |
|  |  | Events × Time | 1, 420 | 0.326 | 0.568 | 0.001 |
|  |  | Group × Events × Time | 3, 420 | 0.086 | 0.968 | 0.001 |
| Communicationhers | p | Time | 1, 420 | 0.148 | 0.701 | 0.000 |
|  |  | Group × Time | 3, 420 | 1.802 | 0.146 | 0.013 |
|  |  | Events × Time | 1, 420 | 0.845 | 0.358 | 0.002 |
|  |  | Group × Events × Time | 3, 420 | 0.768 | 0.512 | 0.005 |
|  | n | Time | 1, 420 | 0.014 | 0.906 | 0.000 |
|  |  | Group × Time | 3, 420 | 1.975 | 0.117 | 0.014 |
|  |  | Events × Time | 1, 420 | 0.921 | 0.338 | 0.002 |
|  |  | Group × Events × Time | 3, 420 | 0.168 | 0.918 | 0.001 |

***Note.*** . **, *p*<.01; *, *p*<.05; Ev, emotional valence; ea, emotional arousal; p, positive events; n, negative, events; NS, non-social contexts; S, social contexts; Following WM training, participants exhibited a significant decrease in negative anticipatory emotion (n-ev, *p* = .005). Significant decreases were observed in Event specificity for both positive (p = .025) and negative events (p = .002), as well as in the Self-relevance of negative simulations (p = .007). No significant Interaction effects were detected.

**Supplementary Table S7-4. LMM analysis of social affective forecasting with continuous trait scores.**

| Metrics | | Source | *df_num_,df_den_* | *F* | *p* | *η^2^_p_* |
| --- | --- | --- | --- | --- | --- | --- |
| Anticipated emotion | p-ev | CSAS × Time | 1, 426 | 1.733 | 0.189 | 0.004 |
|  |  | CSAS × Condition × Time | 1, 426 | 1.790 | 0.182 | 0.004 |
|  |  | BDI × Time | 1, 426 | 2.390 | 0.123 | 0.006 |
|  |  | BDI × Condition × Time | 1, 426 | 0.229 | 0.632 | 0.001 |
|  |  | PHQ × Time | 1, 426 | 2.993 | 0.084 | 0.007 |
|  |  | PHQ × Condition × Time | 1, 426 | 0.353 | 0.553 | 0.001 |
|  | n-ev | CSAS × Time | 1, 426 | 0.567 | 0.452 | 0.001 |
|  |  | CSAS × Condition × Time | 1, 426 | 1.598 | 0.207 | 0.004 |
|  |  | BDI × Time | 1, 426 | 1.879 | 0.171 | 0.004 |
|  |  | BDI × Condition × Time | 1, 426 | 0.060 | 0.807 | 0.000 |
|  |  | PHQ × Time | 1, 426 | 0.061 | 0.805 | 0.000 |
|  |  | PHQ × Condition × Time | 1, 426 | 0.328 | 0.567 | 0.001 |
|  | p-ea | CSAS × Time | 1, 426 | 3.338 | 0.068 | 0.008 |
|  |  | CSAS × Condition × Time | 1, 426 | 0.281 | 0.597 | 0.001 |
|  |  | BDI × Time | 1, 426 | 2.943 | 0.087 | 0.007 |
|  |  | BDI × Condition × Time | 1, 426 | 0.058 | 0.810 | 0.000 |
|  |  | PHQ × Time | 1, 426 | 1.459 | 0.228 | 0.003 |
|  |  | PHQ × Condition × Time | 1, 426 | 0.084 | 0.772 | 0.000 |
|  | n-ea | CSAS × Time | 1, 426 | 0.173 | 0.678 | 0.000 |
|  |  | CSAS × Condition × Time | 1, 426 | 0.342 | 0.559 | 0.001 |
|  |  | BDI × Time | 1, 426 | 0.255 | 0.614 | 0.001 |
|  |  | BDI × Condition × Time | 1, 426 | 3.015 | 0.083 | 0.007 |
|  |  | PHQ × Time | 1, 426 | 0.278 | 0.598 | 0.001 |
|  |  | PHQ × Condition × Time | 1, 426 | 2.168 | 0.142 | 0.005 |
| Anticipatory emotion | p-ev | CSAS × Time | 1, 426 | 0.832 | 0.362 | 0.002 |
|  |  | CSAS × Condition × Time | 1, 426 | 4.172 | 0.042* | 0.010 |
|  |  | BDI × Time | 1, 426 | 1.292 | 0.256 | 0.003 |
|  |  | BDI × Condition × Time | 1, 426 | 0.106 | 0.745 | 0.000 |
|  |  | PHQ × Time | 1, 426 | 0.338 | 0.561 | 0.001 |
|  |  | PHQ × Condition × Time | 1, 426 | 0.046 | 0.830 | 0.000 |
|  | n-ev | CSAS × Time | 1, 426 | 1.631 | 0.202 | 0.004 |
|  |  | CSAS × Condition × Time | 1, 426 | 1.660 | 0.198 | 0.004 |
|  |  | BDI × Time | 1, 426 | 0.437 | 0.509 | 0.001 |
|  |  | BDI × Condition × Time | 1, 426 | 0.196 | 0.658 | 0.000 |
|  |  | PHQ × Time | 1, 426 | 0.159 | 0.691 | 0.000 |
|  |  | PHQ × Condition × Time | 1, 426 | 0.074 | 0.786 | 0.000 |
|  | p-ea | CSAS × Time | 1, 426 | 1.184 | 0.277 | 0.003 |
|  |  | CSAS × Condition × Time | 1, 426 | 0.010 | 0.922 | 0.000 |
|  |  | BDI × Time | 1, 426 | 0.890 | 0.346 | 0.002 |
|  |  | BDI × Condition × Time | 1, 426 | 1.192 | 0.276 | 0.003 |
|  |  | PHQ × Time | 1, 426 | 0.583 | 0.446 | 0.001 |
|  |  | PHQ × Condition × Time | 1, 426 | 0.393 | 0.531 | 0.001 |
|  | n-ea | CSAS × Time | 1, 426 | 0.456 | 0.500 | 0.001 |
|  |  | CSAS × Condition × Time | 1, 426 | 0.309 | 0.578 | 0.001 |
|  |  | BDI × Time | 1, 426 | 0.539 | 0.463 | 0.001 |
|  |  | BDI × Condition × Time | 1, 426 | 2.101 | 0.148 | 0.005 |
|  |  | PHQ × Time | 1, 426 | 2.641 | 0.105 | 0.006 |
|  |  | PHQ × Condition × Time | 1, 426 | 0.128 | 0.721 | 0.000 |
| Motivation | expected effort | CSAS × Time | 1, 426 | 3.682 | 0.056 | 0.009 |
|  |  | CSAS × Condition × Time | 1, 426 | 1.355 | 0.245 | 0.003 |
|  |  | BDI × Time | 1, 426 | 3.172 | 0.076 | 0.007 |
|  |  | BDI × Condition × Time | 1, 426 | 1.619 | 0.204 | 0.004 |
|  |  | PHQ × Time | 1, 426 | 1.383 | 0.240 | 0.003 |
|  |  | PHQ × Condition × Time | 1, 426 | 2.063 | 0.152 | 0.005 |
| Sensory | p | CSAS × Time | 1, 426 | 0.006 | 0.940 | 0.000 |
|  |  | CSAS × Condition × Time | 1, 426 | 0.012 | 0.911 | 0.000 |
|  |  | BDI × Time | 1, 426 | 4.397 | 0.037* | 0.010 |
|  |  | BDI × Condition × Time | 1, 426 | 0.061 | 0.806 | 0.000 |
|  |  | PHQ × Time | 1, 426 | 1.439 | 0.231 | 0.003 |
|  |  | PHQ × Condition × Time | 1, 426 | 0.673 | 0.412 | 0.002 |
|  | n | CSAS × Time | 1, 426 | 0.781 | 0.377 | 0.002 |
|  |  | CSAS × Condition × Time | 1, 426 | 0.084 | 0.771 | 0.000 |
|  |  | BDI × Time | 1, 426 | 0.841 | 0.360 | 0.002 |
|  |  | BDI × Condition × Time | 1, 426 | 0.215 | 0.643 | 0.001 |
|  |  | PHQ × Time | 1, 426 | 0.015 | 0.901 | 0.000 |
|  |  | PHQ × Condition × Time | 1, 426 | 0.000 | 0.993 | 0.000 |
| Event | p | CSAS × Time | 1, 426 | 4.888 | 0.028* | 0.011 |
|  |  | CSAS × Condition × Time | 1, 426 | 0.114 | 0.736 | 0.000 |
|  |  | BDI × Time | 1, 426 | 2.433 | 0.120 | 0.006 |
|  |  | BDI × Condition × Time | 1, 426 | 0.422 | 0.516 | 0.001 |
|  |  | PHQ × Time | 1, 426 | 2.211 | 0.138 | 0.005 |
|  |  | PHQ × Condition × Time | 1, 426 | 0.001 | 0.976 | 0.000 |
|  | n | CSAS × Time | 1, 426 | 9.451 | 0.002** | 0.022 |
|  |  | CSAS × Condition × Time | 1, 426 | 1.020 | 0.313 | 0.002 |
|  |  | BDI × Time | 1, 426 | 4.871 | 0.028* | 0.011 |
|  |  | BDI × Condition × Time | 1, 426 | 0.110 | 0.741 | 0.000 |
|  |  | PHQ × Time | 1, 426 | 1.325 | 0.250 | 0.003 |
|  |  | PHQ × Condition × Time | 1, 426 | 0.001 | 0.973 | 0.000 |
| Self | p | CSAS × Time | 1, 426 | 0.285 | 0.594 | 0.001 |
|  |  | CSAS × Condition × Time | 1, 426 | 0.129 | 0.720 | 0.000 |
|  |  | BDI × Time | 1, 426 | 1.249 | 0.264 | 0.003 |
|  |  | BDI × Condition × Time | 1, 426 | 0.700 | 0.403 | 0.002 |
|  |  | PHQ × Time | 1, 426 | 0.573 | 0.449 | 0.001 |
|  |  | PHQ × Condition × Time | 1, 426 | 0.141 | 0.707 | 0.000 |
|  | n | CSAS × Time | 1, 426 | 3.319 | 0.069 | 0.008 |
|  |  | CSAS × Condition × Time | 1, 426 | 3.709 | 0.055 | 0.009 |
|  |  | BDI × Time | 1, 426 | 0.020 | 0.886 | 0.000 |
|  |  | BDI × Condition × Time | 1, 426 | 0.764 | 0.383 | 0.002 |
|  |  | PHQ × Time | 1, 426 | 0.002 | 0.961 | 0.000 |
|  |  | PHQ × Condition × Time | 1, 426 | 2.659 | 0.104 | 0.006 |
| Others | p | CSAS × Time | 1, 426 | 0.089 | 0.766 | 0.000 |
|  |  | CSAS × Condition × Time | 1, 426 | 2.834 | 0.093 | 0.007 |
|  |  | BDI × Time | 1, 426 | 2.634 | 0.105 | 0.006 |
|  |  | BDI × Condition × Time | 1, 426 | 3.368 | 0.067 | 0.008 |
|  |  | PHQ × Time | 1, 426 | 1.194 | 0.275 | 0.003 |
|  |  | PHQ × Condition × Time | 1, 426 | 1.601 | 0.206 | 0.004 |
|  | n | CSAS × Time | 1, 426 | 0.010 | 0.920 | 0.000 |
|  |  | CSAS × Condition × Time | 1, 426 | 0.147 | 0.701 | 0.000 |
|  |  | BDI × Time | 1, 426 | 2.492 | 0.115 | 0.006 |
|  |  | BDI × Condition × Time | 1, 426 | 0.057 | 0.811 | 0.000 |
|  |  | PHQ × Time | 1, 426 | 0.200 | 0.655 | 0.000 |
|  |  | PHQ × Condition × Time | 1, 426 | 0.028 | 0.867 | 0.000 |
| Communication | p | CSAS × Time | 1, 426 | 0.848 | 0.358 | 0.002 |
|  |  | CSAS × Condition × Time | 1, 426 | 0.894 | 0.345 | 0.002 |
|  |  | BDI × Time | 1, 426 | 0.179 | 0.673 | 0.000 |
|  |  | BDI × Condition × Time | 1, 426 | 3.419 | 0.065 | 0.008 |
|  |  | PHQ × Time | 1, 426 | 0.029 | 0.864 | 0.000 |
|  |  | PHQ × Condition × Time | 1, 426 | 1.832 | 0.177 | 0.004 |
|  | n | CSAS × Time | 1, 426 | 0.576 | 0.448 | 0.001 |
|  |  | CSAS × Condition × Time | 1, 426 | 0.142 | 0.706 | 0.000 |
|  |  | BDI × Time | 1, 426 | 0.186 | 0.667 | 0.000 |
|  |  | BDI × Condition × Time | 1, 426 | 0.638 | 0.425 | 0.001 |
|  |  | PHQ × Time | 1, 426 | 0.420 | 0.517 | 0.001 |
|  |  | PHQ × Condition × Time | 1, 426 | 0.597 | 0.440 | 0.001 |

***Note.*** **, *p*<.01; *, *p*<.05; A significant CSAS × Condition × Time interaction was found for positive anticipatory emotion (*p*=0.042), with further analysis revealed that individual with high social anhedonia exhibited decreased positive anticipatory emotion in non-social events (*p*=.007) whereas no significant change was observed for the social events (*p* = 0.913) or with individuals with low or moderate social anhedonia (*p*>.05). Training-induced decreases in Event specificity were moderated by social anhedonia. Specifically, for positive events, the decrease was significant only in the individuals with high social anhedonia (*p* = .022); for negative events, the decrease was significant for both average (*p* = .007) and high social anhedonia (*p* < .001) individuals.

**Supplementary Table S8-1. Updates of belief because of news in belief updating task.**

|  |  | News | Positive events | Negative events |
| --- | --- | --- | --- | --- |
| SA(N=29) | pre | good | 5.48(4.08) | 4.38(4.89) |
|  |  | bad | 5.55(4.65) | 4.23(3.1) |
|  |  |  |  |  |
|  | post | good | 5.44(3.56) | 4.62(4.59) |
|  |  | bad | 4.01(3.55) | 5.08(3.25) |
|  |  |  |  |  |
| SD(N=40) | pre | good | 7.23(5.28) | 5.27(5.17) |
|  |  | bad | 5.33(5.14) | 5.59(4.67) |
|  |  |  |  |  |
|  | post | good | 6.06(4.37) | 4.89(4.17) |
|  |  | bad | 5.4(4.78) | 5.89(3.81) |
|  |  |  |  |  |
| CO(N=36) | pre | good | 5.55(5.46) | 5.98(6) |
|  |  | bad | 4.44(4.56) | 5.65(4.56) |
|  |  |  |  |  |
|  | post | good | 5.97(5.06) | 6.52(7.33) |
|  |  | bad | 5.85(5.27) | 5.77(4.43) |
|  |  |  |  |  |
| CN(N=38) | pre | good | 5.23(3.81) | 6.07(4.68) |
|  |  | bad | 4.48(3.78) | 5.7(4.21) |
|  |  |  |  |  |
|  | post | good | 7.06(5.39) | 5.11(5.33) |
|  |  | bad | 4.97(3.91) | 5.33(5.52) |
|  |  |  |  |  |
| *F_Group_* | | | 0.465 | 0.913 |
| *p* | | | 0.707 | 0.436 |
| *η*^2^*_p_* | | | 0.010 | 0.019 |
| *F_Time_* | | | 0.344 | 0.020 |
| *p* | | | 0.559 | 0.888 |
| *η*^2^*_p_* | | | 0.002 | <.001 |
| *F_News_* | | | 7.491 | 0.018 |
| *p* | | | **0.007** | 0.894 |
| *η*^2^*_p_* | | | 0.051 | <.001 |
| *F_Group×Time_* | | | 2.484 | 0.786 |
| *p* | | | **0.063** | 0.503 |
| *η*^2^*_p_* | | | 0.051 | 0.017 |
| *F_Group×News_* | | | 0.325 | 0.482 |
| *p* | | | 0.807 | 0.696 |
| *η*^2^*_p_* | | | 0.007 | 0.010 |
| *F_News×Time_* | | | 0.068 | 0.304 |
| *p* | | | 0.794 | 0.582 |
| *η*^2^*_p_* | | | <.001 | 0.002 |

***Note.*** Pre, results before the WM training; Post, results after the WM training. In belief updating task, 29 participants in SA group, 40 participants in SD group, 36 participants in CO group and 38 participants in CN group completed both pre- and post- training measurement.

**Supplementary Table S8-2. LMM analysis of belief updating across groups.**

| Metrics | Source | *df_num_,df_den_* | *F* | *p* | *η^2^_p_* |
| --- | --- | --- | --- | --- | --- |
| positive_update | Time | 1, 426 | 0.219 | 0.640 | 0.001 |
|  | Group × Time | 3, 426 | 2.117 | 0.097 | 0.015 |
|  | news × Time | 1, 426 | 0.020 | 0.888 | 0.000 |
|  | Group × news × Time | 3, 426 | 1.127 | 0.338 | 0.008 |
| negative_update | Time | 1, 426 | 0.116 | 0.734 | 0.000 |
|  | Group × Time | 3, 426 | 0.922 | 0.430 | 0.006 |
|  | news × Time | 1, 426 | 0.438 | 0.509 | 0.001 |
|  | Group × news × Time | 3, 426 | 0.183 | 0.908 | 0.001 |

***Note.*** No significant fixed effects regarding time were observed in BU task.

**Supplementary Table S8-3. LMM analysis of belief updating with continuous trait scores.**

| Metrics | Source | *df_num_,df_den_* | *F* | *p* | *η^2^_p_* |
| --- | --- | --- | --- | --- | --- |
| positive_update | CSAS × Time | 1, 432 | 0.074 | 0.786 | 0.000 |
|  | CSAS × Condition × Time | 1, 432 | 0.991 | 0.320 | 0.002 |
|  | BDI × Time | 1, 432 | 0.284 | 0.594 | 0.001 |
|  | BDI × Condition × Time | 1, 432 | 3.996 | 0.046* | 0.009 |
|  | PHQ × Time | 1, 432 | 0.052 | 0.820 | 0.000 |
|  | PHQ × Condition × Time | 1, 432 | 4.185 | 0.041* | 0.010 |
| negative_update | CSAS × Time | 1, 432 | 1.434 | 0.232 | 0.003 |
|  | CSAS × Condition × Time | 1, 432 | 0.908 | 0.341 | 0.002 |
|  | BDI × Time | 1, 432 | 3.247 | 0.072 | 0.007 |
|  | BDI × Condition × Time | 1, 432 | 0.468 | 0.494 | 0.001 |
|  | PHQ × Time | 1, 432 | 3.078 | 0.080 | 0.007 |
|  | PHQ × Condition × Time | 1, 432 | 0.858 | 0.355 | 0.002 |

**Note.** *, p<.05; Although the results revealed significant three-way interactions for positive belief updating (BDI: p = .046; PHQ: p = .041), follow-up simple slope analyses did not find significant pre-to-post changes within specific trait levels.

**Supplementary Table S9-1. The adaptive intention to pay effort and adaptive consummatory pleasure of reward in the effort expenditure for reward task.**

|  |  |  | Decision | Consummatory pleasure | Decision_slope | Consummatory_slope | beta_decision | beta_consummatory |
| --- | --- | --- | --- | --- | --- | --- | --- | --- |
| SA(N=31) | pre | small | 0.46(0.37) | 6.09(1.02) | 0.21(0.36) | 0.35(0.76) | 0.15(0.32) | 0.2(0.52) |
|  |  | large | 0.54(0.35) | 6.51(1.06) | 0.06(0.09) | 0.19(0.29) |  |  |
|  | post | small | 0.61(0.39) | 6.22(1) | 0.17(0.37) | 0.19(0.71) | 0.11(0.32) | 0.03(0.39) |
|  |  | large | 0.66(0.36) | 6.67(0.88) | 0.06(0.09) | 0.26(0.34) |  |  |
| SD(N=41) | pre | small | 0.68(0.27) | 6.35(1.12) | 0.26(0.4) | 0.31(0.79) | 0.18(0.37) | 0.1(0.25) |
|  |  | large | 0.71(0.28) | 6.51(1.15) | 0.08(0.1) | 0.22(0.28) |  |  |
|  | post | small | 0.64(0.28) | 6.14(1.18) | 0.32(0.41) | 0.49(0.72) | 0.24(0.34) | 0.09(0.49) |
|  |  | large | 0.76(0.22) | 6.82(0.98) | 0.08(0.09) | 0.29(0.29) |  |  |
| CO(N=37) | pre | small | 0.59(0.35) | 6.11(1.02) | 0.36(0.45) | 0.53(1) | 0.25(0.39) | -0.06(1.22) |
|  |  | large | 0.79(0.2) | 6.61(0.86) | 0.11(0.11) | 0.29(0.32) |  |  |
|  | post | small | 0.61(0.31) | 6.1(1.08) | 0.4(0.41) | 0.32(0.84) | 0.3(0.34) | 0.02(0.34) |
|  |  | large | 0.81(0.23) | 6.55(0.95) | 0.11(0.13) | 0.27(0.33) |  |  |
| CN(N=41) | pre | small | 0.69(0.28) | 6.15(1.02) | 0.34(0.38) | 0.26(0.6) | 0.26(0.34) | 0.14(0.32) |
|  |  | large | 0.78(0.25) | 6.66(1.14) | 0.09(0.1) | 0.26(0.33) |  |  |
|  | post | small | 0.65(0.32) | 6.06(1.12) | 0.24(0.37) | 0.22(0.59) | 0.16(0.36) | -0.01(0.48) |
|  |  | large | 0.78(0.25) | 6.96(0.95) | 0.08(0.12) | 0.3(0.27) |  |  |
| *F_Group_* | | | 3.648 | 0.220 | 2.003 | 0.641 | 1.299 | 0.746 |
| *p* | | | **0.014** | 0.882 | 0.116 | 0.590 | 0.277 | 0.526 |
| *η*^2^*_p_* | | | 0.070 | 0.005 | 0.040 | 0.013 | 0.026 | 0.015 |
| *F_Time_* | | | 3.419 | 1.077 | 0.156 | 0.027 | 0.024 | 0.876 |
| *p* | | | **0.066** | 0.301 | 0.693 | 0.869 | 0.876 | 0.351 |
| *η*^2^*_p_* | | | 0.023 | 0.007 | 0.001 | <.001 | <.001 | 0.006 |
| *F_Range_* | | | 29.259 | 45.205 | 73.039 | 2.877 |  |  |
| *p* | | | **0.000** | **0.000** | **0.000** | **0.092** |  |  |
| *η*^2^*_p_* | | | 0.167 | 0.236 | 0.333 | 0.019 |  |  |
| *F_Group×Time_* | | | 3.099 | 0.379 | 1.131 | 1.404 | 1.488 | 0.739 |
| *p* | | | **0.029** | 0.768 | 0.339 | 0.244 | 0.220 | 0.530 |
| *η*^2^*_p_* | | | 0.060 | 0.008 | 0.023 | 0.028 | 0.030 | 0.015 |
| *F_Group×Range_* | | | 2.086 | 0.844 | 1.314 | 1.170 |  |  |
| *p* | | | 0.105 | 0.472 | 0.272 | 0.323 |  |  |
| *η*^2^*_p_* | | | 0.041 | 0.017 | 0.026 | 0.023 |  |  |
| *F_Range×Time_* | | | 0.489 | 2.916 | 0.028 | 1.854 |  |  |
| *p* | | | 0.485 | **0.090** | 0.867 | 0.175 |  |  |
| *η*^2^*_p_* | | | 0.003 | 0.020 | <.001 | 0.013 |  |  |

***Note.*** Decision, the proportion of decisions on high-effort tasks; Consummatory pleasure, the rating scores of the rating when then chosen tasks were completed; Decision_slope, the slope of the linear model that described the proportion of high-effort task choices that change as the reward value changes.; Consummatory_slope, the slope of the linear model that described the consummatory pleasure ratings that change as the reward value changes; Beta_decision, the variance of decision slopes between the small reward amplitude and the large reward amplitude; Beta_consummatory, the variance of consummatory slopes between the small reward amplitude and the large reward amplitude. Pre, results before the WM training; Post, results after the WM training. In EEfRT, 31 participants in SA group, 41 participants in SD group, 37 participants in CO group and 41 participants in CN group completed both pre- and post- training measurement.

**Supplementary Table S9-2. LMM analysis of the EEfRT across groups.**

| Metrics | Source | *df_num_,df_den_* | *F* | *p* | *η^2^_p_* |
| --- | --- | --- | --- | --- | --- |
| Decision_slope | Time | 1, 438 | 0.145 | 0.704 | 0.000 |
|  | Group × Time | 3, 438 | 1.007 | 0.389 | 0.007 |
|  | Condition × Time | 1, 438 | 0.014 | 0.907 | 0.000 |
|  | Group × Condition × Time | 3, 438 | 0.934 | 0.424 | 0.006 |
| Consummatory_slope | Time | 1, 438 | 0.033 | 0.856 | 0.000 |
|  | Group × Time | 3, 438 | 1.567 | 0.197 | 0.011 |
|  | Condition × Time | 1, 438 | 1.545 | 0.215 | 0.004 |
|  | Group × Condition × Time | 3, 438 | 0.900 | 0.441 | 0.006 |
| Decision | Time | 1, 438 | 3.170 | 0.076 | 0.007 |
|  | Group × Time | 3, 438 | 2.874 | 0.036* | 0.019 |
|  | Condition × Time | 1, 438 | 0.436 | 0.509 | 0.001 |
|  | Group × Condition × Time | 3, 438 | 0.438 | 0.726 | 0.003 |
| Consummatory pleasure | Time | 1, 438 | 0.921 | 0.338 | 0.002 |
|  | Group × Time | 3, 438 | 0.324 | 0.808 | 0.002 |
|  | Condition × Time | 1, 438 | 2.689 | 0.102 | 0.006 |
|  | Group × Condition × Time | 3, 438 | 1.039 | 0.375 | 0.007 |
| beta_decision | Time | 1, 146 | 0.022 | 0.882 | 0.000 |
|  | Group × Time | 3, 146 | 1.508 | 0.215 | 0.030 |
| beta_consummatory | Time | 1, 292 | 0.877 | 0.350 | 0.003 |
|  | Group × Time | 3, 292 | 0.745 | 0.526 | 0.008 |

***Note.*** *, *p*<.05; The results revealed a significant Group × Time interaction (*p* = .036). Follow-up simple slope analysis (comparing pre- vs. post-training) showed that only the SA group exhibited a significant increase in high-effort choices (*p* = .002). No significant changes were observed in the healthy control group or other risk groups (p > .05).

**Supplementary Table S9-3. LMM analysis of EEfRT with continuous trait scores.**

| Metrics | Source | *df_num_,df_den_* | *F* | *p* | *η^2^_p_* |
| --- | --- | --- | --- | --- | --- |
| Decision_slope | CSAS × Time | 1, 444 | 0.000 | 0.996 | 0.000 |
|  | CSAS × Condition × Time | 1, 444 | 0.014 | 0.905 | 0.000 |
|  | BDI × Time | 1, 444 | 2.995 | 0.084 | 0.007 |
|  | BDI × Condition × Time | 1, 444 | 3.561 | 0.060^a^ | 0.008 |
|  | PHQ × Time | 1, 444 | 0.524 | 0.470 | 0.001 |
|  | PHQ × Condition × Time | 1, 444 | 0.898 | 0.344 | 0.002 |
| Consummatory_slope | CSAS × Time | 1, 444 | 1.971 | 0.161 | 0.004 |
|  | CSAS × Condition × Time | 1, 444 | 1.184 | 0.277 | 0.003 |
|  | BDI × Time | 1, 444 | 0.155 | 0.694 | 0.000 |
|  | BDI × Condition × Time | 1, 444 | 0.204 | 0.652 | 0.000 |
|  | PHQ × Time | 1, 444 | 0.990 | 0.320 | 0.002 |
|  | PHQ × Condition × Time | 1, 444 | 0.504 | 0.478 | 0.001 |
| Decision | CSAS × Time | 1, 444 | 3.480 | 0.063^a^ | 0.008 |
|  | CSAS × Condition × Time | 1, 444 | 0.524 | 0.469 | 0.001 |
|  | BDI × Time | 1, 444 | 0.263 | 0.608 | 0.001 |
|  | BDI × Condition × Time | 1, 444 | 0.013 | 0.911 | 0.000 |
|  | PHQ × Time | 1, 444 | 0.890 | 0.346 | 0.002 |
|  | PHQ × Condition × Time | 1, 444 | 0.024 | 0.878 | 0.000 |
| Consummatory pleasure | CSAS × Time | 1, 444 | 1.259 | 0.262 | 0.003 |
|  | CSAS × Condition × Time | 1, 444 | 1.631 | 0.202 | 0.004 |
|  | BDI × Time | 1, 444 | 0.123 | 0.726 | 0.000 |
|  | BDI × Condition × Time | 1, 444 | 0.070 | 0.792 | 0.000 |
|  | PHQ × Time | 1, 444 | 0.432 | 0.511 | 0.001 |
|  | PHQ × Condition × Time | 1, 444 | 0.007 | 0.934 | 0.000 |
| beta_decision | CSAS × Time | 1, 148 | 0.023 | 0.880 | 0.000 |
|  | BDI × Time | 1, 148 | 5.861 | 0.017* | 0.038 |
|  | PHQ × Time | 1, 148 | 1.453 | 0.230 | 0.010 |
| beta_consummatory | CSAS × Time | 1,296 | 1.083 | 0.299 | 0.004 |
|  | BDI × Time | 1,296 | 0.910 | 0.341 | 0.003 |
|  | PHQ × Time | 1296 | 1.315 | 0.253 | 0.009 |

***Note.*** *a, p*<.10; *, p<.05; Although the CSAS × Time interaction was marginally significant (*p* = .063), follow-up simple slope analysis revealed a significant training effect in individuals with high CSAS who showed a significant increase in high-effort choices (*p* = .019); Also, LMM analysis revealed a significant BDI × Time interaction for the beta parameter (*p* = .017). Follow-up simple slope analysis showed that while no specific level of BDI reached statistical significance (*p*>.05).

**Supplementary Table S10-1. Adaptive wanting or liking in Effort Reward Imbalance Task.**

|  |  |  | Wanting | Liking | Beta_wanting | Beta_liking |
| --- | --- | --- | --- | --- | --- | --- |
| SA(N=26) | pre | Average | 4.86(1.31) | 5.27(0.88) | 0.5(0.34) | 0.77(0.19) |
|  |  | Top | 5.24(1.51) | 6.11(0.79) |  |  |
|  |  | Bottom | 3.08(1.39) | 2.34(1.28) |  |  |
|  | post | Average | 4.99(1.23) | 5.36(0.77) | 0.61(0.29) | 0.84(0.17) |
|  |  | Top | 5.52(1.31) | 6.24(0.68) |  |  |
|  |  | Bottom | 3.2(1.44) | 2.43(1.2) |  |  |
| SD(N=37) | pre | Average | 4.84(1.07) | 5.45(0.87) | 0.48(0.26) | 0.8(0.2) |
|  |  | Top | 5.21(1.21) | 6.21(0.84) |  |  |
|  |  | Bottom | 3.44(1.14) | 2.31(0.84) |  |  |
|  | post | Average | 4.95(1.05) | 5.39(0.99) | 0.58(0.26) | 0.84(0.21) |
|  |  | Top | 5.43(1.05) | 6.18(0.97) |  |  |
|  |  | Bottom | 3.35(1.36) | 2.42(0.99) |  |  |
| CO(N=34) | pre | Average | 4.32(1.3) | 5.03(0.82) | 0.45(0.35) | 0.76(0.24) |
|  |  | Top | 4.39(1.69) | 5.79(1.13) |  |  |
|  |  | Bottom | 2.74(1.16) | 2.17(0.78) |  |  |
|  | post | Average | 4.54(1.26) | 4.87(1.04) | 0.54(0.29) | 0.75(0.25) |
|  |  | Top | 4.83(1.45) | 5.69(1.19) |  |  |
|  |  | Bottom | 2.68(1.32) | 2.27(0.95) |  |  |
| CN(N=37) | pre | Average | 4.71(1.24) | 5.13(0.88) | 0.51(0.35) | 0.81(0.17) |
|  |  | Top | 4.96(1.56) | 6.13(0.81) |  |  |
|  |  | Bottom | 2.79(1.11) | 2.17(0.82) |  |  |
|  | post | Average | 4.82(1.01) | 5.07(0.91) | 0.59(0.31) | 0.81(0.19) |
|  |  | Top | 5.16(1.1) | 5.87(0.93) |  |  |
|  |  | Bottom | 2.85(1.38) | 2.16(0.81) |  |  |
| *F_Group_* | | | 2.683 | 2.755 | 0.345 | 0.798 |
| *p* | | | **0.049** | **0.045** | 0.793 | 0.497 |
| *η*^2^*_p_* | | | 0.058 | 0.06 | 0.008 | 0.018 |
| *F_Time_* | | | 4.384 | 0.111 | 13.450 | 3.017 |
| *p* | | | **0.038** | 0.74 | **0.000** | **0.085** |
| *η*^2^*_p_* | | | 0.033 | 0.001 | 0.094 | 0.023 |
| *F_Ratio_* | | | 287.643 | 907.839 |  |  |
| *p* | | | **0.000** | **0.000** |  |  |
| *η*^2^*_p_* | | | 0.689 | 0.875 |  |  |
| *F_Group×Time_* | | | 0.147 | 1.108 | 0.030 | 1.044 |
| *p* | | | 0.931 | 0.348 | 0.993 | 0.375 |
| *η*^2^*_p_* | | | 0.003 | 0.025 | 0.001 | 0.024 |
| *F_Group×Ratio_* | | | 0.861 | 0.459 |  |  |
| *p* | | | 0.473 | 0.747 |  |  |
| *η*^2^*_p_* | | | 0.019 | 0.010 |  |  |
| *F_Ratio×Time_* | | | 4.842 | 1.350 |  |  |
| *p* | | | **0.019** | 0.258 |  |  |
| *η*^2^*_p_* | | | 0.036 | 0.01 |  |  |

***Note.*** T, effort-reward balance, ratio =1; A, effort < reward imbalance, ratio >1; B, effort > reward imbalance, ratio < 1. Pre, results before the WM training; Post, results after the WM training. In ERI, 26 participants in SA group, 37 participants in SD group, 34 participants in CO group and 37 participants in CN group completed both pre- and post- training measurement.

**Supplementary Table S10-2. LMM analysis of the ERI across groups**

| Metrics | Source | *df_num_,df_den_* | *F* | *p* | *η^2^_p_* |
| --- | --- | --- | --- | --- | --- |
| Wanting | Time | 1, 655 | 5.863 | 0.016* | 0.009 |
|  | Group × Time | 3, 655 | 0.227 | 0.877 | 0.001 |
|  | Condition × Time | 2, 655 | 1.631 | 0.197 | 0.005 |
|  | Group × Condition × Time | 6, 655 | 0.148 | 0.990 | 0.001 |
| Liking | Time | 1, 655 | 0.060 | 0.806 | 0.000 |
|  | Group × Time | 3, 655 | 0.624 | 0.600 | 0.003 |
|  | Condition × Time | 2, 655 | 0.586 | 0.557 | 0.002 |
|  | Group × Condition × Time | 6, 655 | 0.192 | 0.979 | 0.002 |
| Beta_Wanting | Time | 1, 131 | 13.411 | <.001*** | 0.093 |
|  | Group × Time | 3, 131 | 0.041 | 0.989 | 0.001 |
| Beta_Liking | Time | 1, 131 | 2.999 | 0.086 | 0.022 |
|  | Group × Time | 3, 131 | 1.127 | 0.341 | 0.025 |

***Note.*** ***, *p*<.001; The adaptability of wanting significantly increased following training across all groups (*p*<.001).

**Supplementary Table S10-3. LMM analysis of ERI with continuous trait scores.**

| Metrics | Source | *df_num_,df_den_* | *F* | *p* | *η^2^_p_* |
| --- | --- | --- | --- | --- | --- |
| Wanting | CSAS × Time | 1, 665 | 0.543 | 0.461 | 0.001 |
|  | CSAS × Condition × Time | 2, 665 | 0.085 | 0.918 | 0.000 |
|  | BDI × Time | 1, 665 | 0.035 | 0.851 | 0.000 |
|  | BDI × Condition × Time | 2, 665 | 0.262 | 0.770 | 0.001 |
|  | PHQ × Time | 1, 665 | 0.779 | 0.378 | 0.001 |
|  | PHQ × Condition × Time | 2, 665 | 1.742 | 0.176 | 0.005 |
| Liking | CSAS × Time | 1, 665 | 0.064 | 0.800 | 0.000 |
|  | CSAS × Condition × Time | 2, 665 | 0.167 | 0.846 | 0.001 |
|  | BDI × Time | 1, 665 | 1.106 | 0.293 | 0.002 |
|  | BDI × Condition × Time | 2, 665 | 0.157 | 0.855 | 0.000 |
|  | PHQ × Time | 1, 665 | 2.862 | 0.091 | 0.004 |
|  | PHQ × Condition × Time | 2, 665 | 0.711 | 0.492 | 0.002 |
| Beta_Wanting | CSAS × Time | 1, 133 | 0.000 | 0.987 | 0.000 |
|  | BDI × Time | 1, 133 | 0.024 | 0.878 | 0.000 |
|  | PHQ × Time | 1, 133 | 1.087 | 0.299 | 0.008 |
| Beta_Liking | CSAS × Time | 1, 133 | 0.025 | 0.875 | 0.000 |
|  | BDI × Time | 1, 133 | 0.110 | 0.741 | 0.001 |
|  | PHQ × Time | 1, 133 | 3.779 | 0.054 | 0.028 |

***Note.*** No significant interaction effects regarding time and trait scores were observed in ERI task.

**Supplementary Table S11-1. Accuracy of performance in the sustained attention to response task.**

|  |  | RT | Hit | Commission error |
| --- | --- | --- | --- | --- |
| SA(N=31) | pre | 337.48(58.44) | 0.98(0.04) | 0.5(0.19) |
|  | post | 342.94(45.08) | 0.96(0.17) | 0.45(0.19) |
| SD(N=41) | pre | 321.5(60.28) | 0.97(0.07) | 0.54(0.22) |
|  | post | 339.48(68.47) | 0.97(0.11) | 0.47(0.20) |
| CO(N=34) | pre | 304.02(49.22) | 0.98(0.05) | 0.57(0.20) |
|  | post | 323.91(53.53) | 0.98(0.04) | 0.54(0.20) |
| CN(N=42) | pre | 325.19(48.51) | 0.98(0.03) | 0.51(0.21) |
|  | post | 353.52(69.57) | 0.97(0.06) | 0.46(0.21) |
| *F_Group_* | | 1.841 | 0.205 | 1.231 |
| *p* | | 0.142 | 0.893 | 0.301 |
| *η*^2^*_p_* | | 0.037 | 0.004 | 0.025 |
| *F_Time_* | | 22.880 | 0.692 | 10.262 |
| *p* | | **0.000** | 0.407 | **0.002** |
| *η*^2^*_p_* | | 0.137 | 0.005 | 0.067 |
| *F_Group×Time_* | | 1.515 | 0.607 | 0.266 |
| *p* | | 0.213 | 0.612 | 0.850 |
| *η*^2^*_p_* | | 0.031 | 0.012 | 0.006 |

***Note.*** RT, reaction time; Hit, the proportion of trials that correct respond; Correct rejection, the proportion of trials that reject to respond when targets presented. Pre, results before the WM training; Post, results after the WM training. As for the sustained attention to response task, 31 participants in SA group, 41 participants in SD group, 35 participants in CO group and 41 participants in CN group were involved in both pre- and post- training measurements.

**Supplementary Table S11-2. Discrimination and adaptability in SART**

|  |  | d_prime | In_beta |
| --- | --- | --- | --- |
| SA(N=13) | pre | 1.87(0.77) | -2.15(0.85) |
|  | post | 2.32(0.9) | -2.58(0.91) |
| SD(N=20) | pre | 1.65(0.97) | -1.84(1.12) |
|  | post | 1.85(0.85) | -2.07(1.04) |
| CO(N=15) | pre | 1.62(1.06) | -1.95(1.34) |
|  | post | 1.79(0.89) | -2.15(1.11) |
| CN(N=21) | pre | 1.83(0.89) | -2.08(0.91) |
|  | post | 1.91(0.96) | -1.96(1.04) |
| *F_Group_* | | 0.664 | 0.613 |
| *p* | | 0.577 | 0.609 |
| *η*^2^*_p_* | | 0.030 | 0.028 |
| *F_Time_* | | 4.967 | 1.752 |
| *p* | | **0.029** | 0.190 |
| *η*^2^*_p_* | | 0.071 | 0.026 |
| *F_Group×Time_* | | 0.540 | 0.703 |
| *p* | | 0.657 | 0.554 |
| *η*^2^*_p_* | | 0.024 | 0.031 |

***Note.*** d-prime, representing the ability to discriminate between Go and No-Go stimuli; In_beta, representing the response bias.

**Supplementary Table S11-3. LMM analysis of SART acorss groups.**

| Metrics | Source | *df_num_,df_den_* | *F* | *p* | *η^2^_p_* |
| --- | --- | --- | --- | --- | --- |
| RT | Time | 1, 144 | 22.751 | <.001*** | 0.136 |
|  | Group × Time | 3, 144 | 1.513 | 0.214 | 0.031 |
| Hit | Time | 1, 144 | 0.723 | 0.396 | 0.005 |
|  | Group × Time | 3, 144 | 0.588 | 0.624 | 0.012 |
| Commission error | Time | 1, 143 | 10.196 | 0.002** | 0.067 |
|  | Group × Time | 3, 143 | 0.225 | 0.879 | 0.005 |
| d_prime | Time | 1, 93 | 5.192 | 0.025* | 0.053 |
|  | Group × Time | 3, 93 | 0.776 | 0.510 | 0.024 |
| In_beta | Time | 1, 105 | 1.677 | 0.198 | 0.016 |
|  | Group × Time | 3, 105 | 1.290 | 0.282 | 0.036 |

***Note. .*** ***, *p*<.001; **, p<.01; *, p<.05; Across all groups, participants showed significant universal improvements in sustained attention following training: RT significantly decreased (*p* < .001); Commission errors significantly decreased (*p* = .002); d prime

significantly increased (*p* = .025).

**Supplementary Table S11-4. LMM analysis of SART with continuous trait scores.**

| Metrics | Source | *df_num_,df_den_* | *F* | *p* | *η^2^_p_* |
| --- | --- | --- | --- | --- | --- |
| RT | CSAS × Time | 1, 146 | 1.300 | 0.256 | 0.009 |
|  | BDI × Time | 1, 146 | 0.002 | 0.966 | 0.000 |
|  | PHQ × Time | 1, 146 | 0.026 | 0.873 | 0.000 |
| Hit | CSAS × Time | 1, 146 | 0.176 | 0.675 | 0.001 |
|  | BDI × Time | 1, 146 | 0.064 | 0.800 | 0.000 |
|  | PHQ × Time | 1, 146 | 0.037 | 0.847 | 0.000 |
| Commission error | CSAS × Time | 1, 145 | 0.195 | 0.659 | 0.001 |
|  | BDI × Time | 1, 145 | 0.077 | 0.782 | 0.001 |
|  | PHQ × Time | 1, 145 | 0.814 | 0.368 | 0.006 |
| d_prime | CSAS × Time | 1, 98 | 0.433 | 0.512 | 0.004 |
|  | BDI × Time | 1, 96 | 0.213 | 0.645 | 0.002 |
|  | PHQ × Time | 1, 96 | 1.248 | 0.267 | 0.013 |
| In_beta | CSAS × Time | 1, 110 | 1.294 | 0.258 | 0.012 |
|  | BDI × Time | 1, 108 | 0.047 | 0.828 | 0.000 |
|  | PHQ × Time | 1, 110 | 0.006 | 0.938 | 0.000 |

***Note.*** No significant interaction effects regarding time and trait scores were observed in SART.

**Supplementary Table S12. Summary of WM training effects.**

|  |  |  | **Timepoint main effect** | **Interaction effect with Timepoint** |
| --- | --- | --- | --- | --- |
| **Hedonic processing** | | | | |
|  | **Reward processing** | | | |
|  | The Monetary Incentives Task | Reaction time | Significant, post < pre |  |
|  |  | Anticipatory pleasure | Significant, post < pre | Condition * timepoint, significant: decreased in reward condition |
|  |  | Consummatory pleasure when hit the target | Significant, post < pre | Condition * timepoint, significant: difference between punishment and neutral condition turned to be not significant after training |
|  |  | Consummatory pleasure when miss the target |  | Condition * timepoint, significant: increased in punishment condition |
|  | The Social Incentives Task | Reaction time | Significant, post < pre |  |
|  |  | Anticipatory pleasure | Significant, post < pre | Condition * timepoint, significant: decreased in punishment condition |
|  |  | Consummatory pleasure when hit the target | Significant, post < pre | Condition * timepoint, significant: difference between punishment and neutral condition turned to be significant after training |
|  |  | Consummatory pleasure when miss the target | Significant, post > pre |  |
|  | **Prospection** | | | |
|  | The Social Affective Forecasting Task | Anticipated valence |  |  |
|  |  | Anticipated arousal |  |  |
|  |  | Anticipatory valence | Significant for negative events, post > pre | Condition * timepoint, marginally significant for positive events: decreased in nonsocial context |
|  |  | Anticipatory arousal |  | Condition * timepoint, significant for negative events: difference between social and nonsocial contexts turned to be not significant after training |
|  |  | Self-reported motivation to engage in positive events |  |  |
|  |  | Self-rated details of the imaginings |  |  |
|  |  | 1)sensory details |  |  |
|  |  | 2)event details | Significant for negative events, post < pre |  |
|  |  | 3) self-referential thoughts; | Significant for both negative and positive events, post < pre | Condition * timepoint, significant for positive events: decreased in nonsocial context |
|  |  | 4) other-referential thoughts |  |  |
|  |  | 5) communications |  |  |
|  | The Belief Updating Task | Belief Updating Score |  | Group * timepoint, marginally significant for positive events: increased in control group |
|  | **Cost-benefit computation** | | | |
|  | The Effort Expenditure for Reward Task | Decision proportion on high effort tasks | Marginally significant, post > pre | Group * timepoint, significant: increased in group of high social anhedonia |
|  |  | Consummatory pleasure rating after complete the chosen task |  | Condition * timepoint, marginally significant: increased in large reward range |
|  |  | Range adaptability for reward on decision proportion |  |  |
|  |  | Range adaptability for reward on consummatory pleasure rating |  |  |
|  | The Effort Reward Imbalance Task | Wanting ratings of reward | Significant, post >pre | Condition * timepoint, significant: increased in condition that reward is more than effort |
|  |  | Liking ratings of reward |  |  |
|  |  | Adaptability for imbalance on wanting ratings | Significant, post > pre |  |
|  |  | Adaptability for imbalance on liking ratings | Marginally significant, post > pre |  |
| **Cognitive functions** | | | | |
|  | **Working memory capacity** | | | |
|  | Dual N-Back Task | N-max | Significant, post > pre |  |
|  |  | N-average | Significant, post > pre |  |
|  | Letter Number Span Task | LNS-max | Significant, post > pre |  |
|  |  | LNS-sum | Significant, post > pre |  |
|  | **Executive function** | | | |
|  | The Sustained Attention to Response Task | Hit rate |  |  |
|  |  | Commission Error | Significant, post < pre |  |

***Note.*** Pre, assessments before WM training; Post, assessments after training; Condition * timepoint, interaction effect of condition by timepoint; Group * timepoint, interaction effect of Group by timepoint. Blank row represented for no significant results.
